# Design and synthesis of multivalent neoglycoconjugates by click conjugations

**DOI:** 10.3762/bjoc.10.134

**Published:** 2014-06-10

**Authors:** Feiqing Ding, Li Ji, Ronny William, Hua Chai, Xue-Wei Liu

**Affiliations:** 1 Division of Chemistry and Biological Chemistry, School of Physical and Mathematical Sciences, Nanyang Technological University, 21 Nanyang Link, Singapore 637371

**Keywords:** click conjugations, copper-catalyzed, microwave irradiation, multivalent glycosystems, neoglycoconjugates, one-pot

## Abstract

A highly stereoselective BF_3_∙OEt_2_-promoted tandem hydroamination/glycosylation on glycal scaffolds has been developed to form propargyl 3-tosylamino-2,3-dideoxysugars in a one-pot manner. Subsequent construction of multivalent 3-tosylamino-2,3-dideoxyneoglycoconjugates with potential biochemical applications was presented herein involving click conjugations as the key reaction step. The copper-catalyzed regioselective click reaction was tremendously accelerated with assistance of microwave irradiation.

## Introduction

Oligosaccharides and glycopeptides are the key constituents of the cellular membrane and extracellular matrix, and play a pivotal role in various key cellular events such as cell–cell recognition, host–pathogen or host–symbiont interactions, molecular recognition of antibodies and metastasis [1–5]. The construction of a 1,4-disubstituted-1,2,3-triazole unit via a copper(I)-catalyzed modern version of the Huisgen-type azide–alkyne cycloaddition [6–10] has been considered to be a powerful ligation method for glycoconjugation [11–16]. In addition to the simplicity of this reaction and the ease of purification, 1,4-disubstituted-1,2,3-triazoles, the regiospecific product of this reaction, exhibit similarities to the ubiquitous amide moiety found in nature. However, unlike amides, the triazole moiety proved to be robust and resistant to chemical and enzymatic cleavage [17–20]. Moreover, the inertness of both azide and alkyne groups towards a majority of functional groups connected to the core of a variety of biomolecules also renders the click reaction particularly suitable for covalently linking bioactive molecular entities [21–22]. For example, the click strategy is especially versatile for the effective construction of complex glycosylated structures such as clusters, dendrimers, polymers, peptides and macrocycles. In all the cases the triazole ring plays a crucial role in combining divergent units together to establish a complex molecular architecture [23–31].

**Figure 1:**
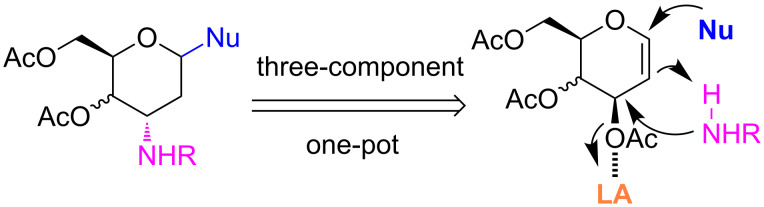
Our reported strategy for quick access to 3-amino-2,3-dideoxysugars via regio- and stereoselective tandem hydroamination/glycosylation of glycals.

**Figure 2:**
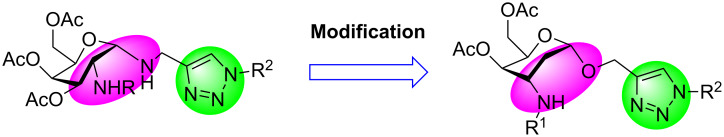
Synthetic modification of α-GalNAc linked glycopeptides to 3-tosylamino-2,3-dideoxyneoglycoconjugates via click conjugation.

The α-GalNAc-linked glycopeptides, α-*N*-glycosidically linked to the polypeptide chain through the amido nitrogen of an asparagine residue at the *N*-terminal [32], were found to be the most important semi-synthetic glycoconjugates, usually modified from their naturally occurring parent precursors [33–39]. Over the years, many structural analogues of this class of antibiotics have been synthesized. In addition, triazoles are considered as peptidic linkage surrogates. Surprisingly, despite the enormous research interests associated with their synthesis, only a few examples of oligosaccharides and glycopeptides mimics have so far been prepared by a click chemistry strategy [40–48]. Most recently, we developed a strategy for the synthesis of 3-amino-2,3-dideoxysugars using a regio- and stereoselective tandem hydroamination/glycosylation of the glycal shown in Figure 1 [49–53]. Extending the synthetic utility of this protocol, herein, we wish to report the synthetic modification of α-GalNAc-linked glycopeptides to 3-tosylamino-2,3-dideoxyneoglycoconjugates via click conjugations (Figure 2).

**Figure 3:**
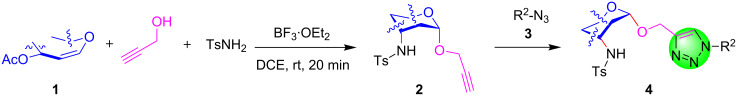
Our proposal for access to 3-tosylamino-2,3-dideoxyneoglycoconjugates via tandem hydroamination/glycosylation of glycals followed by click conjugations.

Given the success in using “click chemistry” in the glycosylation reactions, we aspired to apply the highly efficient triazole formation employing an azide **3** and a suitable alkyne appended to the 3-amino-2,3-dideoxysugars moiety **2** (Figure 3). In continuation of our previous work, herein we report a direct and reliable synthetic approach to multivalent 3-tosylamino-2,3-dideoxyneoglyco conjugates **4** with potential biochemical applications involving click conjugations as the key reaction step (Figure 3).

## Results and Discussion

**Scheme 1:**
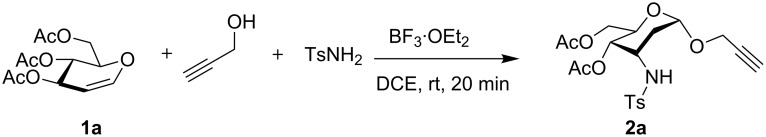
Synthesis of propargyl 3-tosylamino-2,3-dideoxy-α-D-allohexopyranoside (**2a**).

Primarily, we successfully synthesized propargyl 3-*p*-toluenesulfonamido-4,6-di-*O*-acetyl-2,3-dideoxy-α-D-allohexopyranoside (**2a**) in gram scale via BF_3_∙OEt_2_-promoted one-pot three-component α-selective tandem hydroamination/glycosylation reaction (Scheme 1). In fact, when 3,4,6-tri-*O*-acetyl-D-glucal (**1a**), propargyl alcohol and *p*-toluenesulfonamide were subjected to a one-pot reaction in the presence of 2.2 equiv of BF_3_∙OEt_2_ in DCE at room temperature for 20 min, the desired aminoglycoside **2a** was obtained in good yield with exclusive α-stereoselectivity [50]. Later, a systematic screening was executed using 3-tosylamino-2,3-dideoxysugar **2a** and benzyl azide (**3a**) as our model system under varied conditions of catalysts, additives, solvents and reaction temperatures (Table 1). The initial evaluation involved no catalyst and additives at 100 °C and DMF, MeCN/H_2_O 3:1 or MeOH as the solvent system, which resulted in unsuccessful reactions (Table 1, entries 1–3). However, a trace amount of the desired product was detected in the presence of 10 mol % of copper(I) iodide (Table 1, entry 4). The combination of CuSO_4_·5H_2_O (10 mol %) and sodium ascorbate (10 mol %) was found to be a suitable catalyst leading regiospecifically to the 1,4-disubstituted-1,2,3-triazole **4a** with moderate yield of 46% in *t*-BuOH/H_2_O 1:1 after 20 hours at 70 °C (Table 1, entry 5). The yield was further improved to 97% by employing DMF as solvent in a shorter period of 12 hours (Table 1, entry 6). Encouraged by these results, we attempted to improve the assemblies and to shorten the reaction times further; reactions were subjected to microwave irradiation, which is best known to accelerate transition metal-catalyzed homogeneous reactions [54]. Microwave-assisted organic reactions are rapidly becoming recognized as a valuable tool for facilitating a wide variety of organic transformations [55–56]. Finally, we found that the rate of conversion accelerated dramatically when microwave irradiation was used under 70 °C. To our delight under microwave conditions and in DMF with addition of 1 mol % of CuSO_4_·5H_2_O and 10 mol % of sodium ascorbate, a quantative yield of desired 3-tosylamino-2,3-dideoxyneoglycoconjugate **4a** was obtained in 15 min (Table 1, entry 7).

**Table 1:** Optimization for synthesis of 3-tosylamino-2,3-dideoxyneoglycoconjugate **4a**.

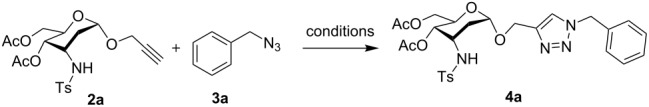					

Entry	Catalyst (mol %)	Solvent	Temperature (°C)	Time (h)	Yield (mol %)^a^

1	none	DMF	100	20	NR^b^
2	none	MeCN/H_2_O	100	20	NR^b^
3	none	MeOH	100	20	NR^b^
4	CuI (10)	THF	60	12	trace
5	CuSO_4_·5H_2_O (1)	*t-*BuOH/H_2_O	70	20	46
6	CuSO_4_·5H_2_O (1)	DMF	70	12	97
7	CuSO_4_·5H_2_O (1)	DMF	70^c^	0.25	98

^a^Isolated yield after purification. ^b^NR = no reaction. ^c^Assisted by microwave irradiation, 200 W.

Next, the required α-propargyl 3-tosylamino-2,3-dideoxyglycosides **2** were synthesized by BF_3_∙OEt_2_-promoted one-pot three-component tandem hydroamination/glycosylation reaction on a glycal scaffold including tri-*O*-acetyl-D-glucal (**1a**), tri-*O*-acetyl-D-allal (**1b**), tri-*O*-acetyl-D-galactal (**1c**), di-*O*-acetyl-D-rhamnal (**1d**), hexa-*O*-acetyl-D-maltal (**1e**). Accordingly, a series of α-propargyl 3-tosylamino-2,3-dideoxyglycosides **2a**–**2d** were obtained exclusively with α-stereoselectivity in good yields (Table 2, entries 1–5).

**Table 2:** One-pot synthesis of α-propargyl 3-tosylamino-2,3-dideoxyglycosides **2**.

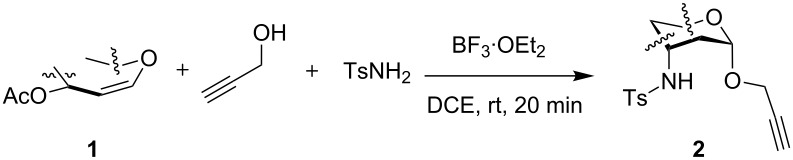			

Entry	**1**	**2**	Yield (%)^a^

1	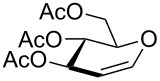	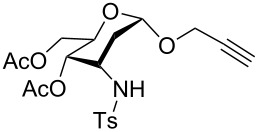	86
	**1a**	**2a**	
2	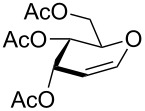	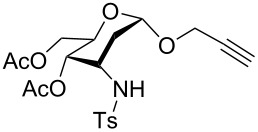	84
	**1b**	**2a**	
3	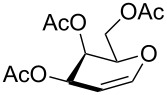	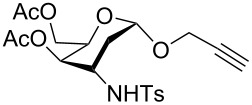	81
	**1c**	**2b**	
4	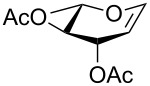	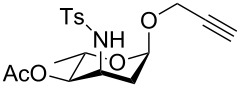	74
	**1d**	**2c**	
5	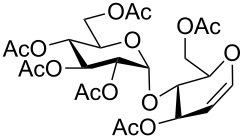	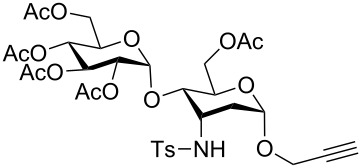	67
	**1e**	**2d**	

^a^Isolated yields after purification.

With pure α-propargyl 3-tosylamino-2,3-dideoxyglycosides and the optimized conditions in hand, we focused on performing a Huisgen cycloaddition reaction. The scope and generality of this method to prepare 3-tosylamino-2,3-dideoxyneoglycoconjugates **4** with the assistance of copper sulfate and sodium ascorbate was examined extensively. A range of α-alkyne-3-tosylamino-2,3-dideoxysugars and azides with various substituent groups (R^2^) were screened and the summarized results are shown in Table 3. Overall, the yields obtained were from good to excellent while preserving the anomeric selectivity and regioselectivity. In general, the analogous reaction of a set of azides with different substituent groups (**3a**–**3e**) with α-propargyl 3-tosylamino-2,3-dideoxy glycosides **2** afforded the corresponding 3-tosylamino-2,3-dideoxyneoglycoconjugates (**4a**–**4h)** in good to excellent yields with exclusive anomeric selectivity (Table 3, entries 1–8). This encouraging result prompted us to apply these conditions to alkyne **2a** and a series of azido-linked monosaccharides **3f**, **3g** and **3h** as well as the propargyl disaccharide **2d** with α-GlaNAc azido **3g** which were also obtained in good yields and selectivities (Table 3, entries 9–13). Subsequently, to shorten the reaction times, we subjected all the click conjugations to microwave irradiation. All the reactions were completed in considerably shorter reaction times of less than 30 min for the Huisgen cycloaddition of alkenes and azides catalyzed by copper sulfate and sodium ascorbate, affording the corresponding products in good to excellent yields in each case (Table 3, method B). This result showed that the synthesis of 3-tosylamino-2,3-dideoxyneoglycoconjugates via copper-catalyzed Huisgen cycloaddition is highly efficient under microwave irradiation.

**Table 3:** Scope for synthesis of 3-tosylamino-2,3-dideoxyneoglycoconjugates.

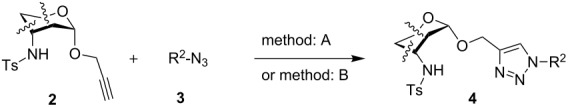					

Entry	**2**	**3**	**4**	Yield (%)^a^	

				A^b^	B^c^

1	**2a**	**3a**	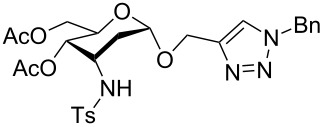	97	98
			**4a**		
2	**2b**	**3a**	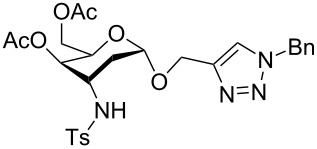	89	93
			**4b**		
3	**2c**	**3a**	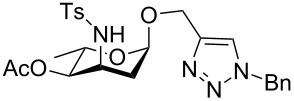	74	81
			**4c**		
4	**2d**	**3a**	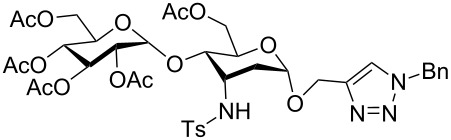	71	78
			**4d**		
5	**2a**	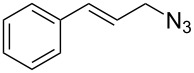	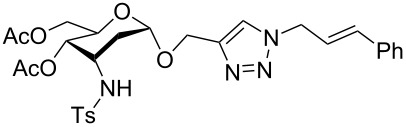	82	85
		**3b**	**4e**		
6	**2a**	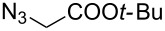	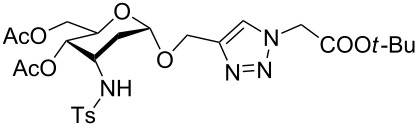	91	92
		**3c**	**4f**		
7	**2a**	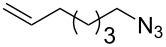	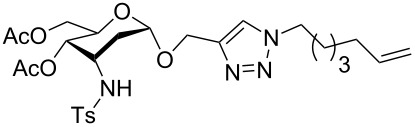	86	89
		**3d**	**4g**		
8	**2a**	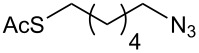	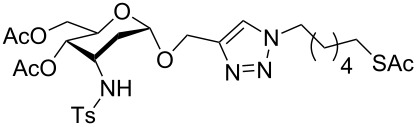	87	92
		**3e**	**4h**		
9	**2a**	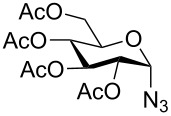	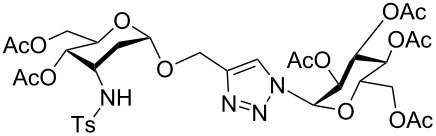	76	80
		**3f**	**4i**		
10	**2a**	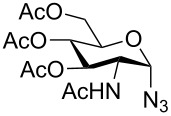	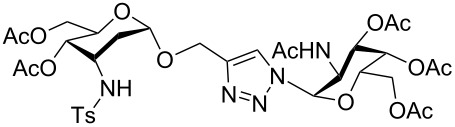	93	95
		**3g**	**4j**		
11	**2d**	**3g**	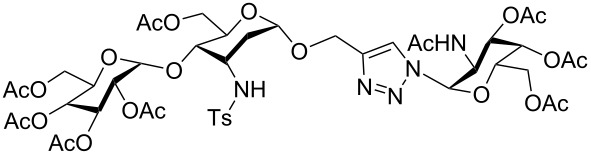	80	82
			**4k**		
12	**2a**	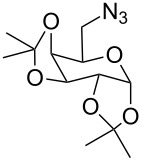	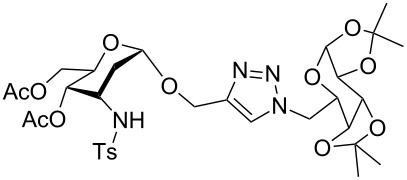	72	78
		**3h**	**4l**		

^a^Isolated yields after purification. ^b^70 °C under conventional heating, 12 hours. ^c^70 °C under microwave irradiation, 200 W, 15 minutes.

**Scheme 2:**
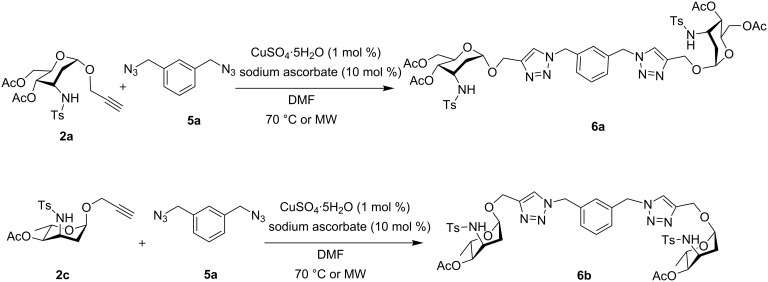
Synthesis of divalent 3-tosylamino-2,3-dideoxyneoglycoconjugates **6a** and **6b**.

**Scheme 3:**
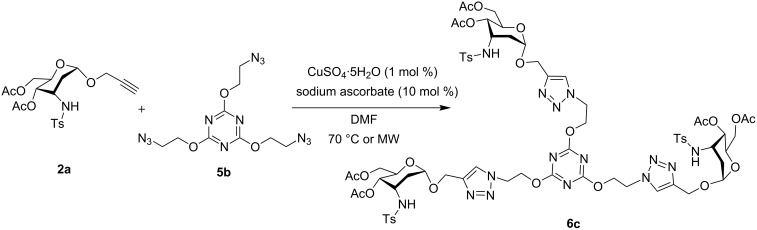
Synthesis of trivalent 3-tosylamino-2,3-dideoxyneoglycoconjugate **6c**.

In carbohydrate recognition events, higher multivalent interactions are absolutely essential as the monovalent affinities of carbohydrate monosaccharides are comparatively low and weak. To enhance this multivalent effect, thereby increasing the binding efficiencies of carbohydrates with the coupling counterparts, there has been a constant development of new glycoconjugates such as glycodendrimers [57]. Hence, as continuation of previous encouraging results, we have further designed the use of noncarbohydrate diazide **5a** in the cycloaddition reaction with the α-propargyl 3-tosylamino-2,3-dideoxyalloside **2a** and α-propargyl 3-tosylamino-2,3,6-trideoxy-α-L-ribohexopyranoside **2c** (Scheme 2) to obtain divalent 3-tosylamino-2,3-dideoxyneoglycoconjugates **6a** and **6b** in 83% and 61% yield respectively. The synthesis of trivalent 3-tosylamino-2,3-dideoxyneoglycoconjugates **6c** was also feasible by using triazide **5b** in 66% yield (Scheme 3). Interestingly, for all the reactions under microwave irradiation, reaction times were reduced to 15 minutes. As such, this novel synthetic protocol provides a straightforward access to a wide range of 3-tosylamino-2,3-dideoxyneoglycoconjugate derivatives which may find numerous biochemical applications [40–48].

## Conclusion

In conclusion, it has been established that the construction of well-defined multivalent, anomerically pure 3-amino-2,3-dideoxyneoglycoconjugate architectures was successfully achieved by using cycloaddition reactions of alkynes and azides. It is expected that this strategy will find extensive applications in glycoscience, because triazole-linked glycoconjugates can exhibit very interesting biological properties, offering a convenient access toward oligosaccharides, glycopeptide mimics, or multivalent carbohydrate systems [40–48]. Their further application in molcecular biosystems is currently underway and the results will be reported in due course.

## Supporting Information

File 1 Experimental, analytical data and ^1^H NMR and ^13^C NMR spectra for all new compounds.
