# Supplementary material for: Design and synthesis of multivalent neoglycoconjugates by click conjugations
Source: Beilstein J Org Chem. 2014 Jun 10;10:1325–32. doi: 10.3762/bjoc.10.134 (PMC4077470; doi:10.3762/bjoc.10.134)
Supplement: File 1 — Experimental, analytical data and 1H NMR and 13C NMR spectra for all new compounds. [file Beilstein_J_Org_Chem-10-1325-s001.pdf]

Supporting Information

for

Design and synthesis of multivalent neoglycoconjugates by click conjugations

Feiqing Ding, Li Ji, Ronny William, Hua Chai, and Xue-Wei Liu *

Address: Division of Chemistry and Biological Chemistry, School of Physical and Mathematical Sciences, Nanyang Technological University, 21 Nanyang Link, Singapore 637371

Email: Xue-Wei Liu * - xuewei@ntu.edu.sg

* Corresponding author

Table of Contents **Page**

Table of Contents	Page
Experimental	S2
General procedure for the synthesis of propagyl 3-tosylamino-2,3-dideoxysugars **2a**–**2d**	S2
Spectroscopic characterization of propagyl 3-tosylamino-2,3-dideoxysugars **2a**–**2d**	S3
General procedures for the synthesis of multivalent 3-tosylamino-2,3-dideoxyneoglycoconjugates	S6
Spectroscopic characterization of multivalent 3-tosylamino-2,3-dideoxyneoglycoconjugates	S6
References	S24
NMR spectra for all compounds	S26

Experimental

All solvents were distilled under nitrogen from the following drying agents immediately before use: tetrahydrofuran (THF) was distilled from sodium/benzophenone ketyl; dichloromethane and 1,2-dichloroethane were distilled from calcium hydride. Anhydrous acetonitrile (MeCN) was purchased from commercial suppliers and used without further purification. $BF_3 \cdot OEt_2$ was distilled from calcium hydride before use. The compounds **1a**, **1d**, **3a**, $TsNH_2$ and propargyl alcohol were purchased from commercial suppliers and used without further purification. The compounds **1b** [1], **1c** [1], **1e** [1], **3a**–**3h** [2-6], and **5a** and **5b** [7-9] were prepared according to literature reported procedures. All the catalysts and additives were purchased from commercial suppliers and used without further purification. High resolution mass spectra (HRMS) were recorded on a Waters Q-Tof premier^{TM} mass spectrometer. Microwave experiments were conducted in a CEM Discover^{TM} system.

General procedure for the synthesis of propagyl 3-tosylamino-2,3-dideoxysugars 2a–2d [10]. To a solution of glycal **1** (1.0 equiv) and *p*-methylbenzenesulfonamide (1.1 equiv) in DCE (0.1 M) was added propagyl alcohol (1.1 equiv) under N_2 atmosphere. $BF_3 \cdot OEt_2$ (2.2 equiv) was then added to this mixture. The reaction mixture was stirred for 20 min at room temperature, quenched with saturated $NaHCO_3$ and subsequently extracted with CH_2Cl_2 (3 × 15 mL). The extract was dried under Na_2SO_4 and

concentrated. The residue was subjected to column chromatography (silica gel, hexane/EtOAc) to obtain pure 3-tosylamino-2,3-dideoxysugars **2**.

Propargyl 3-*p*-toluenesulfonamido-4,6-di-*O*-acetyl-2,3-dideoxy-α-D-allo-hexopyranoside (**2a**): Compound **2a** (gummy liquid, 640 mg, 81% yield) was prepared according to the general procedure for the synthesis of propargyl 3-tosylamino-2,3-dideoxysugars from 3,4,6-tri-*O*-acetyl-D-glucal **1a** (500 mg, 1.8 mmol), *p*-methylbenzenesulfonamide (342 mg, 2.0 mmol), and propargyl alcohol (210 μL, 2.0 mmol) and purified by column chromatography (hexane/ethyl acetate = 5 : 1); ^{1}H NMR ($CDCl_3$, 400MHz): δ 7.72 (d, *J* = 8.0 Hz, 2H), 7.28 (d, *J* = 8.0 Hz, 2H), 5.82 (d, *J* = 8.8 Hz, 1H), 5.05 (d, *J* = 3.2 Hz, 1H), 4.65 (dd, *J* = 10.4, 4.0 Hz, 1H), 4.32 (dd, *J* = 12.0, 4.4 Hz, 1H), 4.09-4.27 (m, 3H), 3.91 (q, *J* = 3.6 Hz, 1H), 3.38-3.40 (m, 1H), 2.46 (s, 1H), 2.42 (s, 3H), 2.08 (s, 3H), 2.04 (s, 3H), 1.87 (dt, *J* = 14.8, 4.0 Hz, 1H), 1.50 (dd, *J* = 18.4, 2.4 Hz, 1H); ^{13}C NMR ($CDCl_3$, 100MHz): δ 170.7, 170.4, 143.4, 137.8, 129.8, 126.9, 95.7, 78.1, 75.5, 66.8, 64.7, 62.6, 54.8, 47.8, 32.7, 21.5, 21.0, 20.8; HRMS (ESI) m/z $[M + Na]^+$ calcd for $C_{20}H_{25}NO_8SNa$ 462.1199, found 462.1201.

Propargyl 3-*p*-toluenesulfonamido-4,6-di-*O*-acetyl-2,3-dideoxy-α-D-galacto-pyranoside (**2b**): Compound **2b** (gummy liquid, 66 mg, 84% yield) was prepared according to the general procedure for the synthesis of propargyl 3-tosylamino-2,3-dideoxysugars from tri-*O*-acetyl-D-galactal **1c** (50 mg, 0.18 mmol), *p*-methylbenzenesulfonamide (34 mg, 0.2 mmol), and propargyl alcohol (21 μL, 0.2 mmol) and purified by column chromatography

(hexane/ethyl acetate = 5:1); ^{1}H NMR ($CDCl_3$, 400MHz): δ 7.79 (d, *J* = 8.4 Hz, 2H), 7.29 (d, *J* = 8.4 Hz, 2H), 5.94 (d, *J* = 8.0 Hz, 1H), 5.10 (d, *J* = 3.6 Hz, 1H), 4.79 (d, *J* = 3.2 Hz, 1H), 4.22-4.32 (m, 3H), 4.00-4.10 (m, 2H), 3.58-3.68 (m, 1H), 2.49 (t, *J* = 2.4 Hz, 1H), 2.42 (s, 3H), 2.07 (s, 3H), 2.06 (s, 3H), 2.03 (dt, *J* = 14.8, 4.0 Hz, 1H), 1.47 (dd, *J* = 14.8, 2.4 Hz, 1H); ^{13}C NMR ($CDCl_3$, 100MHz): δ 170.6, 170.4, 143.6, 137.5, 129.8, 127.1, 95.3, 78.3, 75.4, 67.8, 63.5, 62.7, 54.5, 47.4, 28.4, 21.6, 21.5, 20.8; HRMS (ESI) m/z $[M + Na]^+$ calcd for $C_{20}H_{25}NO_8SNa$ 462.1199, found 462.1195.

Propargyl 3-*p*-toluenesulfonamido-4-*O*-acetyl-2,3,6-trideoxy-α-L-ribo-hexopyranoside (**2c**): Compound **2c** (gummy liquid, 51 mg, 74% yield) was prepared according to the general procedure for the synthesis of propargyl 3-tosylamino-2,3-dideoxysugars from di-*O*-acetyl-D-rhamnal **1d** (39 mg, 0.18 mmol), *p*-methylbenzenesulfonamide (34 mg, 0.2 mmol), and propargyl alcohol (21 μL, 0.2 mmol) and purified by column chromatography (hexane/ethyl acetate = 5:1); ^{1}H NMR ($CDCl_3$, 400MHz): δ 7.72 (d, *J* = 8.0 Hz, 2H), 7.29 (d, *J* = 8.0 Hz, 2H), 5.84 (d, *J* = 8.8 Hz, 1H), 4.97 (d, *J* = 3.6 Hz, 1H), 4.35 (dd, *J* = 10.0, 4.0 Hz, 1H), 4.24 (t, *J* = 2.4 Hz, 2H), 4.02-4.06 (m, 1H), 3.83-3.86 (m, 1H), 2.47 (t, *J* = 2.4 Hz, 1H), 2.41 (s, 3H), 2.08 (s, 3H), 1.83 (dt, *J* = 15.6, 4.0 Hz, 1H), 1.47 (dd, *J* = 14.8, 2.8 Hz, 1H); ^{13}C NMR ($CDCl_3$, 100MHz): δ 170.7, 143.2, 138.0, 129.7, 126.9, 95.4, 78.4, 75.2, 72.3, 62.3, 54.5, 47.8, 33.0, 21.5, 21.0, 17.3; HRMS (ESI) m/z $[M + Na]^+$ calcd for $C_{18}H_{23}NO_6SNa$ 404.1144, found 404.11153.

Propargyl 3-*p*-toluenesulfonamido-4-(2',3',4',6',-tetra-*O*-acetyl-α-D-gluco-pyranoside)-6-*O*-acetyl-2,3-dideoxy-α-D-allopyranoside (**2d**) [10]: Compound **2d** (gummy liquid, 88 mg, 67% yield) was prepared according to the general procedure for the synthesis of propargyl 3-tosylamino-2,3-dideoxysugars from hexa-*O*-acetyl-D-maltal **1e** (101 mg, 0.18 mmol), *p*-methylbenzene sulfonamide (34 mg, 0.2 mmol), and propargyl alcohol (21 μL, 0.2 mmol) and purified by column chromatography (hexane/ethyl acetate = 5:1); analytical data of **2d** were in all respect identical with the published data [10]: ^{1}H NMR ($CDCl_3$, 400MHz): δ 7.71 (d, *J* = 8.0 Hz, 2H), 7.31 (d, *J* = 8.0 Hz, 2H), 5.69 (d, *J* = 10.0 Hz, 1H), 5.42-5.46 (m, 1H), 5.24 (dd, *J* = 10.4, 2.4 Hz, 1H), 5.12 (t, *J* = 10.0 Hz, 1H), 4.93 (d, *J* = 1.6 Hz, 1H), 4.47 (dd, *J* = 12.0, 2.4 Hz, 1H), 4.19-4.33 (m, 3H), 4.09-4.17 (m, 4H), 3.95-3.98 (m, 1H), 3.75 (dd, *J* = 9.6, 4.0 Hz, 1H), 2.49 (t, *J* = 2.4 Hz, 1H), 2.43 (s, 3H), 2.20 (s, 3H), 2.11 (s, 3H), 2.09 (s, 3H), 2.04 (s, 3H), 2.00 (s, 3H), 1.61 (dt, *J* = 14.4, 3.6 Hz, 1H), 1.31 (dd, *J* = 14.8, 2.8 Hz, 1H); ^{13}C NMR ($CDCl_3$, 100 MHz): δ 170.6, 170.4, 170.3, 170.0, 169.6, 143.6, 138.1, 129.9, 126.8, 95.7, 92.1, 78.2, 75.4, 70.0, 68.6, 68.4, 68.1, 66.1, 62.9, 61.7, 60.4, 54.9, 46.2, 31.5, 21.5, 20.9, 20.8, 20.7, 20.6, 14.2; HRMS (ESI) m/z $[M + Na]^+$ calcd for $C_{32}H_{41}NO_{16}SNa$ 750.2044, found 750.2042.

General procedures for the synthesis of multivalent 3-tosylamino-2,3-dideoxyneoglycoconjugates.

Method A: To a propargyl 3-tosylamino-2,3-dideoxysugar **2** (0.05 mmol, 1.0 equiv) and azide **3** (1.5 equiv) in DMF (2 mL) was added $CuSO_4·5H_2O$ solution (1 mol %) and sodium ascorbate solution (10 mol %). The reaction mixture was stirred for 12 hours at 70 °C, quenched with water (10 mL) and subsequently extracted with EA (10 mL × 3). The extract was dried and concentrated. The residue was subjected to column chromatography (silica gel, hexane/EtOAc) to obtain pure 3-tosylamino-2,3-dideoxyneoglycoconjugates **4**.

Method B: To a 3-amino-2,3-dideoxysugar **4** (0.05 mmol, 1.0 equiv) and azide **5** (1.5 equiv) in DMF (2 mL) was added $CuSO_4·5H_2O$ solution (1.0 M) (10 mol %) and sodium ascorbate solution (1.0 M) (10 mol %). The reaction vessel was sealed and irradiated in a microwave reactor (CEM Discover™ system) at a temperature of 70 °C for 15 minutes at a maximum power of 200 W. The reaction mixture was cooled to room temperature and quenched with water (10 mL) and subsequently extracted with EA (10 mL × 3). The extract was dried and concentrated. The residue was subjected to column chromatography (silica gel, hexane/EtOAc) to obtain pure 3-tosylamino-2,3-dideoxyneoglyco conjugates **4.**

1-Benzyl-4-(3'-*p*-toluenesulfonamido-4',6'-di-*O*-acetyl-2',3'-dideoxy-α-D-allo-pyranosyloxymethyl)-1*H*-1,2,3-triazol (**4a**): Compound **4a** (gummy liquid, 27 mg, 97% yield) was prepared according to the general procedure

(method A) for the synthesis of multivalent 3-tosylamino-2,3-dideoxyneoglycoconjugates from propargyl 3-tosylamino-2,3-dideoxysugar **2a** (22 mg, 0.05 mmol), benzyl azide **3a** (10 mg, 0.075 mmol), $CuSO_4 \cdot 5H_2O$ solution (5 μL, 1.0 M), and sodium ascorbate solution (5 μL, 1.0 M) in DMF (2 mL). The crude product was purified by column chromatography (hexane/ethyl acetate = 1:1); compound **4a** (28 mg, 98% yield) was also prepared according to the general procedure (method B) for the synthesis of multivalent 3-tosylamino-2,3-dideoxyneoglycoconjugates from propargyl 3-tosylamino-2,3-dideoxysugar **2a** (gummy liquid, 22 mg, 0.05 mmol), benzyl azide (**3a**, 10 mg, 0.075 mmol), $CuSO_4 \cdot 5H_2O$ solution (5 μL, 1.0 M), and sodium ascorbate solution (5 μL, 1.0 M) in DMF (2 mL) under microwave irradiation. The crude product was purified by column chromatography (hexane/ethyl acetate = 1:1); ^1H NMR ($CDCl_3$, 400MHz): δ 7.63 (d, *J* = 8.0 Hz, 2H), 7.51 (s, 1H) , 7.31-7.41 (m, 5H), 7.24 (d, *J* = 8.4 Hz, 2H), 5.94 (d, *J* = 9.2 Hz, 1H), 5.56 (s, 2H), 4.95 (d, *J* = 7.2 Hz,1H), 4.81 (d, *J* = 12.4 Hz, 1H), 4.60-4.65 (m, 2H), 4.29 (dd, *J* = 12.0, 4.4 Hz, 1H), 4.11-4.17 (m, 2H), 3.88-3.91 (m, 1H), 2.40 (s, 3H), 2.06 (s, 3H), 2.01 (s, 3H), 1.83 (dt, *J* = 9.6, 3.6 Hz, 1H), 1.51 (dd, *J* = 14.8, 2.4 Hz, 1H); ^{13}C NMR ($CDCl_3$, 400MHz): δ 170.7, 170.3, 143.9, 143.3, 137.9, 134.4, 129.7, 129.3, 128.9, 128.2, 126.9, 122.5, 96.3, 66.9, 64.6, 62.6, 60.7, 54.3, 47.9, 32.8, 21.5, 20.9, 20.8; HRMS (ESI) m/z $[M + Na]^+$ calcd for $C_{27}H_{32}N_4O_8SNa$ 595.1839, found 595.1838.

1-Benzyl-4-(3'-*p*-toluenesulfonamido-4',6'-di-*O*-acetyl-2',3'-dideoxy-α-D-galactopyranosyloxymethyl)-1*H*-1,2,3-triazol (**4b**): Compound **4b** (gummy liquid, 25 mg, 89% yield) was prepared according to the general procedure (method A) for the synthesis of multivalent 3-tosylamino-2,3-dideoxyneoglyco conjugates from propargyl 3-tosylamino-2,3-dideoxysugar **2b** (22 mg, 0.05 mmol), benzyl azide (**3a**, 10 mg, 0.075 mmol), $CuSO_4·5H_2O$ solution (5 μL, 1.0 M), and sodium ascorbate solution (5 μL, 1.0 M) in DMF (2 mL), which was purified by column chromatography (hexane/ethyl acetate = 1:1); compound **4b** (26 mg, 93% yield) was also prepared according to the general procedure (method B) for the synthesis of multivalent 3-tosylamino-2,3-dideoxyneoglycoconjugates from propargyl 3-tosylamino-2,3-dideoxy sugar **2b** (gummy liquid, 22 mg, 0.05 mmol), benzyl azide (**3a**, 10 mg, 0.075 mmol), $CuSO_4·5H_2O$ solution (5 μL, 1.0 M), and sodium ascorbate solution (5 μL, 1.0 M) in DMF (2 mL) under microwave irradiation. The crude product was purified by column chromatography (hexane/ethyl acetate = 1:1); ^{1}H NMR ($CDCl_3$, 400MHz): δ 7.73 (d, *J* = 8.4 Hz, 2H), 7.53 (s, 1H), 7.37-7.43 (m, 3H), 7.32 (d, *J* = 6.8 Hz, 2H), 7.28 (d, *J* = 8.0 Hz, 2H), 6.01 (d, *J* = 8.0 Hz, 1H), 5.57 (s, 2H), 5.01 (d, *J* = 3.2 Hz, 1H), 4.82 (d, *J* = 12.4 Hz, 1H), 4.75 (d, *J* = 2.8 Hz, 1H), 4.62 (d, *J* = 12.4 Hz,1H), 4.30 (t, *J* = 6.0 Hz,1H), 3.98-4.07 (m, 2H), 3.59-3.60 (m, 1H), 2.41(s, 3H), 2.06 (s, 3H), 2.04 (d, *J* = 4.0 Hz,1H), 2.01 (s, 3H), 1.47 (d, *J* = 14.8 Hz, 1H); ^{13}C NMR ($CDCl_3$, 400MHz): δ 170.5, 169.5, 144.1, 143.5, 137.6, 134.4, 129.8, 129.3,

128.9, 128.2, 127.1, 122.5, 96.1, 67.7, 63.4, 62.8, 60.5, 54.3, 47.5, 28.6, 21.6, 20.8, 20.7; HRMS (ESI) m/z $[M + Na]^+$ calcd for $C_{27}H_{32}N_4O_8SNa$ 595.1839, found 595.1843.

1-Benzyl-4-(3'-*p*-toluenesulfonamido-4'-*O*-acetyl-2',3',6'-trideoxy-α-L-ribo hexopyranosyloxymethyl)-1*H*-1,2,3-triazol (4c): Compound **4c** (gummy liquid, 17 mg, 74% yield) was prepared according to the general procedure (method A) for the synthesis of multivalent 3-tosylamino-2,3-dideoxyneoglycoconjugates from propargyl 3-tosylamino-2,3-dideoxysugar **2c** (19 mg, 0.05 mmol), benzyl azide (**3a**, 10 mg, 0.075 mmol), $CuSO_4 \cdot 5H_2O$ solution (5 μL, 1.0 M), and sodium ascorbate solution (5 μL, 1.0 M) in DMF (2 mL) The crude product was purified by column chromatography (hexane/ethyl acetate = 1:1); compound **4c** (gummy liquid, 21 mg, 81% yield) was also prepared according to the general procedure (method B) for the synthesis of multivalent 3-tosylamino-2,3-dideoxyneoglycoconjugates from propargyl 3-tosylamino-2,3-dideoxy sugar **2c** (19 mg, 0.05 mmol), benzyl azide (**3a**, 10 mg, 0.075 mmol), $CuSO_4 \cdot 5H_2O$ solution (5 μL, 1.0 M), and sodium ascorbate solution (5 μL, 1.0 M) in DMF (2 mL) under microwave irradiation The crude product was purified by column chromatography (hexane/ethyl acetate = 1:1); ^1H NMR ($CDCl_3$, 400MHz): δ 7.62 (d, *J* = 8.4 Hz, 2H), 7.50 (s,1H), 7.34-7.42 (m, 3H), 7.30-7.33 (m, 2H), 7.23 (d, *J* = 8.0 Hz, 2H), 5.95 (d, *J* = 9.2 Hz, 1H), 5.55 (s, 2H), 4.86 (d, *J* =3.2 Hz, 1H), 4.80 (d, *J* = 12.4 Hz, 1H),4.58 (d, *J* = 12.4

Hz, 1H), 4.32 (dd, *J* = 10.0, 4.0 Hz, 1H),4.02 (dd, *J* = 10.0, 6.0 Hz, 1H), 3.79-3.84 (m, 1H), 2.39 (s, 3H), 2.02 (s, 3H), 1.77 (dt, *J* = 14.8, 4.0 Hz, 1H), 1.47 (dd, *J* = 14.8, 2.0 Hz, 1H), 1.17 (d, *J* = 6.0 Hz, 3H); ^{13}C NMR ($CDCl_3$, 400MHz): δ 170.7, 144.2, 143.2, 138.0, 134.4, 129.7, 129.3, 128.9, 128.2, 126.9, 122.4, 96.2, 72.5, 62.2, 60.6, 54.3, 48.0, 33.1, 21.5, 21.0, 17.4; HRMS (ESI) m/z $[M + Na]^+$ calcd for $C_{25}H_{30}N_4O_6SNa$ 537.1784, found 537.1781.

1-Benzyl-4-(4'-(2'',3'',4'',6''-tetra-O-acetyl-α-D-allopyranoside)-6'-*O*-acetyl -2',3'-dideoxy-α-D-allopyranosyloxymethyl)-1*H*-1,2,3-triazol (**4d**): Compound **4d** (gummy liquid, 30 mg, 71% yield) was prepared according to the general procedure (method A) for the synthesis of multivalent 3-tosylamino-2,3-dideoxy neoglycoconjugates from propargyl 3-tosylamino-2,3-dideoxysugar **2d** (36 mg, 0.05 mmol), benzyl azide (**3a**, 10 mg, 0.075 mmol), $CuSO_4·5H_2O$ solution (5 μL, 1.0 M), and sodium ascorbate solution (5 μL, 1.0 M) in DMF (2 mL). The crude product was purified by column chromatography (hexane/ethyl acetate = 1:1); compound **4d** (gummy liquid, 34 mg, 78% yield) was also prepared according to the general procedure (method B) for the synthesis of multivalent 3-tosylamino-2,3-dideoxyneoglycoconjugates from propargyl 3-tosylamino-2,3-dideoxysugar **2d** (36 mg, 0.05 mmol), benzyl azide **3a** (10 mg, 0.075 mmol), $CuSO_4·5H_2O$ solution (5 μL, 1.0 M), and sodium ascorbate solution (5 μL, 1.0 M) in DMF (2 mL) under microwave irradiation. The crude product was purified by column chromatography (hexane/ethyl acetate = 1:1);

^{1}H NMR ($CDCl_3$, 400MHz): δ 7.64-7.69 (m, 1H), 7.56 (d, *J* = 8.0 Hz, 2H), 7.46-7.50 (m, 2H), 7.37-7.41 (m, 1H), 7.32-7.34 (m, 2H), 7.21 (d, *J* = 8.4 Hz, 2H), 5.77 (d, *J* = 9.6 Hz, 1H), 5.58 (s, 2H), 5.44 (d, *J* = 10.0 Hz, 1H), 5.40 (d, *J* = 4.0 Hz, 1H), 5.24 (dd, *J* = 10.4, 3.6 Hz, 1H), 5.11 (t, *J* = 9.6 Hz, 1H), 4.86 (s, 1H), 4.78 (d, *J* = 12.4 Hz, 1H), 4.55 (d, *J* = 12.0 Hz, 1H), 4.44 (dd, *J* = 12.0, 2.8 Hz, 1H), 4.28 (dd, *J* = 12.0, 4.4 Hz, 1H), 4.21 (dd, *J* = 12.4, 4.0 Hz, 1H), 4.09-4.13 (m, 3H), 3.96 (d, *J* = 10.0 Hz, 1H), 3.84-3.88 (m, 1H), 3.72 (dd, *J* = 9.6, 4.0 Hz, 1H), 2.40 (s, 3H), 2.19 (s, 3H), 2.10 (s, 3H), 2.07 (s, 3H), 2.04 (s, 3H), 2.00 (s, 3H), 1.52-1.56 (m, 1H), 1.32-1.36 (m, 1H); ^{13}C NMR ($CDCl_3$, 400MHz): δ 170.6, 170.4, 170.3, 170.0, 169.5, 143.9, 143.5, 138.0, 134.4, 132.1, 131.9, 129.9, 129.3, 128.2, 126.7, 122.5, 96.5, 92.0, 70.0, 68.7, 68.4, 68.1, 65.9, 62.9, 61.7, 61.1, 54.3, 46.2, 31.5, 21.5, 21.0, 20.8, 20.7, 20.6, 14.2; HRMS (ESI) m/z $[M + Na]^+$ calcd for $C_{39}H_{48}N_4O_{16}SNa$ 883.2684, found 883.2686.

1-Cinnamyl-4-(3'-*p*-toluenesulfonamido-4',6'-di-*O*-acetyl-2',3'-dideoxy-α-D-allopyranosyloxymethyl)-1*H*-1,2,3-triazol (**4e**): Compound **4e** (gummy liquid, 24 mg, 82% yield) was prepared according to the general procedure (method A) for the synthesis of multivalent 3-tosylamino-2,3-dideoxyneoglycoconjugates from propargyl 3-tosylamino-2,3-dideoxysugar **2a** (22 mg, 0.05 mmol), cinnamyl azide (**3b**, 12 mg, 0.075 mmol), $CuSO_4·5H_2O$ solution (5 μL, 1.0 M), and sodium ascorbate solution (5 μL, 1.0 M) in DMF (2 mL). The crude product was purified by

column chromatography (hexane/ethyl acetate = 1:1); compound **4e** (gummy liquid, 26 mg, 85% yield) was also prepared according to the general procedure (method B) for the synthesis of multivalent 3-tosylamino-2,3-dideoxyneoglycoconjugates from propargyl 3-tosylamino-2,3-dideoxy sugar **2a** (22 mg, 0.05 mmol), cinnamyl azide (**3b**, 12 mg, 0.075 mmol), $CuSO_4 \cdot 5H_2O$ solution (5 μL, 1.0 M), and sodium ascorbate solution (5 μL, 1.0 M) in DMF (2 mL) under microwave irradiation. The crude product was purified by column chromatography (hexane/ethyl acetate = 1:1); ^{1}H NMR ($CDCl_3$, 400MHz): δ 7.64 (d, *J* = 10.4 Hz, 3H), 7.41 (d, *J* = 7.2 Hz, 2H), 7.26-7.35 (m, 3H), 7.21 (d, *J* = 8.4 Hz, 2H), 6.73 (d, *J* = 16.0 Hz, 1H), 6.38 (dt, *J* = 16.0, 6.8 Hz, 1H), 5.96 (d, *J* = 8.8 Hz, 1H), 5.17 (d, *J* = 6.8 Hz, 2H), 4.97 (d, *J* = 3.2 Hz, 1H), 4.84 (d, *J* = 12.4, 1H), 4.61-4.66 (m, 2H), 4.30 (dd, *J* = 12.4, 4.8 Hz,1H), 4.18 (d, *J* = 10.4 Hz, 2H), 3.88-3.91(m, 1H), 2.37 (s, 3H), 2.06 (s, 3H), 2.00 (s, 3H), 1.83 (dt, *J* = 14.8, 4.0 Hz, 1H), 1.53 (dd, *J* = 14.8, 2.4 Hz, 1H); ^{13}C NMR ($CDCl_3$, 400MHz): δ 170.7, 170.4, 143.8, 143.3, 137.8, 136.0, 135.4, 129.7, 128.8, 128.7, 126.9, 126.8, 122.4, 121.5, 96.3, 66.9, 64.6, 62.6, 60.7, 52.5, 47.9, 32.9, 21.5, 20.9, 20.8; HRMS (ESI) m/z $[M + Na]^+$ calcd for $C_{29}H_{34}N_4O_8SNa$ 621.1995, found 621.1994.

1-*tert*-Butylacetyl-4-(3'-*p*-toluenesulfonamido-4',6'-di-*O*-acetyl-2',3'-dideoxy-α-D-allopyranosyloxymethyl)-1*H*-1,2,3-triazol (**4f**): Compound **4f** (gummy liquid, 27 mg, 91% yield) was prepared according to the general procedure (method A) for the synthesis of multivalent

3-tosylamino-2,3-dideoxyneoglycoconjugates from propargyl 3-tosylamino-2,3-dideoxysugar **2a** (22 mg, 0.05 mmol), azide **3c** (8 mg, 0.075 mmol), $CuSO_4 \cdot 5H_2O$ solution (5 μL, 1.0 M), and sodium ascorbate solution (5 μL, 1.0 M) in DMF (2 mL). The crude product was purified by column chromatography (hexane/ethyl acetate = 1:1); compound **4f** (gummy liquid, 28 mg, 92% yield) was also prepared according to the general procedure (method B) for the synthesis of multivalent 3-tosylamino-2,3-dideoxyneoglycoconjugates from propargyl 3-tosylamino-2,3-dideoxysugar **2a** (22 mg, 0.05 mmol), azide **3c** (8 mg, 0.075 mmol), $CuSO_4 \cdot 5H_2O$ solution (5 μL, 1.0 M), and sodium ascorbate solution (5 μL, 1.0 M) in DMF (2 mL) under microwave irradiation. The crude product was purified by column chromatography (hexane/ethyl acetate = 1:1); ^{1}H NMR ($CDCl_3$, 400MHz): δ 7.72 (s, 1H), 7.66 (d, *J* = 8.0 Hz, 2H), 7.25 (d, *J* = 8.0 Hz, 2H), 5.96 (d, *J* = 8.8 Hz, 1H), 5.08 (s, 2H), 4.96 (d, *J* = 3.2 Hz, 1H), 4.84 (d, *J* = 12.4 Hz, 1H),4.67 (d, *J* = 12.4 Hz, 1H), 4.63 (dd, *J* = 10.0, 4.0 Hz, 1H), 4.29 (dd, *J* = 12.4, 4.8 Hz, 1H), 4.17 (d, *J* = 10.4 Hz, 2H), 3.87-3.90 (m, 1H), 2.39 (s, 3H), 2.06 (s, 3H), 2.01 (s, 3H), 1.82 (dt, *J* = 14.4, 4.0 Hz, 1H), 1.52 (d, *J* = 2.4 Hz, 1H),1.49 (s, 9H); ^{13}C NMR ($CDCl_3$, 400MHz): δ 170.7, 170.4, 165.2, 143.7, 143.3, 137.9, 129.8, 126.9, 124.2, 96.2, 84.0, 67.0, 64.6, 62.7, 60.6, 51.5, 47.9, 32.8, 28.0, 21.5, 20.9, 20.8; HRMS (ESI) m/z $[M + Na]^+$ calcd for $C_{26}H_{36}N_4O_{10}SNa$ 619.2050, found 619.2053.

1-(Hept-6'-en-1-yl)-4-(3''-*p*-toluenesulfonamido-4'',6''-di-*O*-acetyl-2'',3''-dideoxy-α-D-allopyranosyloxymethyl)-1*H*-1,2,3-triazol (**4g**): Compound **4g**

(gummy liquid, 25 mg, 86% yield) was prepared according to the general procedure (method A) for the synthesis of multivalent 3-tosylamino-2,3-dideoxyneoglyco conjugates from propargyl 3-tosylamino-2,3-dideoxysugar **2a** (22 mg, 0.05 mmol), azide **3d** (11 mg, 0.075 mmol), $CuSO_4 \cdot 5H_2O$ solution (5 μL, 1.0 M), and sodium ascorbate solution (5 μL, 1.0 M) in DMF (2 mL). The crude product was purified by column chromatography (hexane/ethyl acetate = 1:1); compound **4g** (gummy liquid, 26 mg, 89% yield) was also prepared according to the general procedure (method B) for the synthesis of multivalent 3-tosylamino-2,3-dideoxyneoglycoconjugates from propargyl 3-tosylamino-2,3-dideoxysugar **2a** (22 mg, 0.05 mmol), azide **3d** (11 mg, 0.075 mmol), $CuSO_4 \cdot 5H_2O$ solution (5 μL, 1.0 M), and sodium ascorbate solution (5 μL, 1.0 M) in DMF (2 mL) under microwave irradiation. The crude product was purified by column chromatography (hexane/ethyl acetate = 1:1); ^{1}H NMR ($CDCl_3$, 400MHz): δ 7.66 (d, *J* = 8.4 Hz, 2H), 7.57 (s, 1H), 7.27 (d, *J* = 6.8 Hz, 2H), 5.97 (d, *J* = 8.8 Hz, 1H), 5.76-5.79 (m, 1H), 4.93-5.01 (m, 3H), 4.84 (d, *J* = 12.4 Hz, 1H), 4.65 (d, *J* = 12.4 Hz, 2H), 4.38 (t, *J* = 7.2 Hz, 2H), 4.32 (dd, *J* = 12.0, 4.8 Hz, 1H), 4.19 (d, *J* = 11.2 Hz, 2H), 3.88-3.92 (m, 1H), 2.40 (s, 3H), 2.07 (s, 3H), 2.03-2.05 (m, 2H), 2.01 (s, 3H), 1.95 (t, *J* = 7.2 Hz, 2H), 1.85 (dt, *J* = 14.4, 4.0 Hz, 1H), 1.24-1.48 (m, 5H); ^{13}C NMR ($CDCl_3$, 400MHz): δ 170.7, 170.4, 143.4, 143.3, 138.3, 137.8, 129.8, 126.9, 122.4, 114.8, 96.3. 67.0, 64.6,

62.7, 60.7, 50.5, 48.0, 33.4, 32.9, 30.1, 28.2, 25.9, 21.5, 20.9, 20.8; HRMS (ESI) m/z $[M + Na]^+$ calcd for $C_{27}H_{38}N_4O_8SNa$ 601.2308, found 601.2308.

1-(6'-Acetylthiohexyl)-4-(3''-*p*-toluenesulfonamido-4'',6''-di-*O*-acetyl-2'', 3''-dideoxy-α-D-allopyranosyloxymethyl)-1*H*-1,2,3-triazol (**4h**): Compound **4h** (gummy liquid, 28 mg, 87% yield) was prepared according to the general procedure (method A) for the synthesis of multivalent 3-tosylamino-2,3-dideoxyneoglycoconjugates from propargyl 3-tosylamino-2,3-dideoxysugar **2a** (22 mg, 0.05 mmol), azide **3e** (10 mg, 0.075 mmol), $CuSO_4{\cdot}5H_2O$ solution (5 μL, 1.0 M), and sodium ascorbate solution (5 μL, 1.0 M) in DMF (2 mL). The crude product was purified by column chromatography (hexane/ethyl acetate = 1:1); compound **4h** (gummy liquid, 30 mg, 92% yield) was also prepared according to the general procedure (method B) for the synthesis of multivalent 3-tosylamino-2,3-dideoxyneoglycoconjugates from propargyl 3-tosylamino-2,3-dideoxysugar **2a** (22 mg, 0.05 mmol), azide **3e** (10 mg, 0.075 mmol), $CuSO_4{\cdot}5H_2O$ solution (5 μL, 1.0 M), and sodium ascorbate solution (5 μL, 1.0 M) in DMF (2 mL) under microwave irradiation. The crude product was purified by column chromatography (hexane/ethyl acetate = 1:1); ^1H NMR ($CDCl_3$, 400MHz): δ 7.65 (d, *J* = 8.4 Hz, 2H), 7.58 (s, 1H), 7.27 (d, *J* = 8.0 Hz, 2H), 5.97 (d, *J* = 8.8 Hz, 1H), 5.75-5.81 (m, 1H), 4.93-5.01 (m, 3H), 4.84 (d, *J* = 12.4 Hz, 1H), 4.62-4.65 (m, 2H), 4.38 (t, *J* = 7.2 Hz, 2H), 4.30 (dd, *J* = 12.0, 4.8 Hz, 1H), 4.17-4.20 (m, 1H), 3.88-3.91 (m, 1H), 2.40 (s, 3H), 2.07 (s, 3H), 2.03

(s, 3H), 1.99 (s, 3H), 1.95 (t, *J* = 7.2 Hz, 2H), 1.85 (dt, *J* = 14.4, 4.0 Hz, 1H), 1.54-1.64 (m, 3H), 1.38-1.40 (m, 4H); ^{13}C NMR ($CDCl_3$, 400MHz): δ 171.2, 170.7, 170.3, 143.5, 143.4, 137.8, 129.8, 126.9, 122.4, 96.3. 67.0, 64.6, 64.2, 62.6, 60.7, 50.4, 48.0, 32.9, 30.2, 28.4, 26.1, 25.4, 21.5, 21.0, 20.9, 20.8; HRMS (ESI) m/z $[M + Na]^+$ calcd for $C_{28}H_{40}N_4O_9S_2Na$ 663.2134, found 663.2130.

1-(2',3',4',6'-Tetra-*O*-acetyl-α-D-allopyranosyl)-4-(3''-*p*-tosamido-4'',6''-di-*O*-acetyl-2'',3''-dideoxy-α-D-allopyranosyloxymethyl)-1*H*-1,2,3-triazol (**4i**): Compound **4i** (gummy liquid, 31 mg, 76% yield) was prepared according to the general procedure (method A) for the synthesis of multivalent 3-tosylamino-2,3-dideoxyneoglycoconjugates from propargyl 3-tosylamino-2,3-dideoxysugar **2a** (22 mg, 0.05 mmol), azide **3f** (28 mg, 0.075 mmol), $CuSO_4{\cdot}5H_2O$ solution (5 μL, 1.0 M), and sodium ascorbate solution (5 μL, 1.0 M) in DMF (2 mL). The crude product was purified by column chromatography (hexane/ethyl acetate = 1:1); compound **4i** (gummy liquid, 33 mg, 80% yield) was also prepared according to the general procedure (method B) for the synthesis of multivalent 3-tosylamino-2,3-dideoxyneoglycoconjugates from propargyl 3-tosylamino-2,3-dideoxysugar **2a** (22 mg, 0.05 mmol), azide **3f** (28 mg, 0.075 mmol), $CuSO_4{\cdot}5H_2O$ solution (5 μL, 1.0 M), and sodium ascorbate solution (5 μL, 1.0 M) in DMF (2 mL) under microwave irradiation. The crude product was purified by column chromatography (hexane/ethyl acetate = 1:1); ^1H NMR

($CDCl_3$, 400MHz): δ 7.84 (s, 1H), 7.70 (d, *J* = 8.4 Hz, 2H), 7.28 (d, *J* = 8.4 Hz, 2H), 5.92 (dd, *J* = 13.6, 9.2 Hz, 2H), 5.40-5.48 (m, 2H), 5.27 (t, *J* = 9.6 Hz, 1H), 4.92 (d, *J* = 3.2 Hz, 1H), 4.85 (d, *J* = 12.4 Hz, 1H), 4.66 (d, *J* = 12.4 Hz, 2H), 4.31-4.38 (m, 2H), 4.16-4.21 (m, 3H), 4.02-4.04 (m, 1H), 3.90-3.92 (m, 1H), 2.41 (s, 3H), 2.07-2.09 (m, 9H), 2.03-2.05 (m, 9H), 1.85 (dt, *J* = 14.8, 4.0 Hz, 1H), 1.53 (dd, *J* = 10.4, 2.4 Hz, 1H); ^{13}C NMR ($CDCl_3$, 400MHz): δ 170.7, 170.5, 170.3, 169.9, 169.4, 169.0, 144.1, 143.3, 138.0, 129.7, 126.9, 121.2, 96.1, 85.9, 75.3, 72.4, 70.4, 67.7, 66.9, 64.6, 62.7, 61.5, 60.2, 47.9, 32.8, 21.5, 20.9, 20.8, 20.7, 20.5, 20.2, 14.2; HRMS (ESI) m/z $[M + Na]^+$ calcd for $C_{34}H_{44}N_4O_{17}SNa$ 835.2320, found 835.2314.

1-(2’-*N*-Acetyl-3’,4’,6’-tri-*O*-acetyl-α-D-allopyranosyl)-4-(3’’-*p*-toluenesulfonamido-4’’,6’’-di-*O*-acetyl-2’’,3’’-dideoxy-α-D-allopyranosyloxymethyl)-1*H*-1,2,3-triazol (**4j**): Compound **4j** (gummy liquid, 38 mg, 93% yield) was prepared according to the general procedure (method A) for the synthesis of multivalent 3-tosylamino-2,3-dideoxyneoglycoconjugates from propargyl 3-tosylamino-2,3-dideoxysugar **2a** (22 mg, 0.05 mmol), azide **3g** (28 mg, 0.075 mmol), $CuSO_4{\cdot}5H_2O$ solution (5 μL, 1.0 M), and sodium ascorbate solution (5 μL, 1.0 M) in DMF (2 mL). The crude product was purified by column chromatography (hexane/ethyl acetate = 1:1); compound **4j** (gummy liquid, 39 mg, 95% yield) was also prepared according to the general procedure (method B) for the synthesis of multivalent 3-tosylamino-2,3-dideoxyneoglycoconjugates from propargyl

3-tosylamino-2,3-dideoxysugar **2a** (22 mg, 0.05 mmol), azide **3g** (28 mg, 0.075 mmol), $CuSO_4$·$5H_2O$ solution (5 μL, 1.0 M), and sodium ascorbate solution (5 μL, 1.0 M) in DMF (2 mL) under microwave irradiation. The crude product was purified by column chromatography (hexane/ethyl acetate = 1:1); ^{1}H NMR ($CDCl_3$, 400MHz): δ 7.89 (d, *J* = 28.4 Hz, 1H), 5.58 (d, *J* = 8.0 Hz, 2H) , 7.21 (d, *J* = 8.4 Hz, 2H), 6.99 (d, *J* = 8.8 Hz, 1H), 6.72 (d, *J* = 9.2 Hz, 1H), 6.14 (d, *J* = 10.0 Hz, 1H), 5.98 (d, *J* = 9.2 Hz, 1H), 5.51 (t, *J* = 10.4 Hz, 1H), 5.24 (t, *J* = 10.0 Hz, 1H), 5.16 (t, *J* = 9.6 Hz, 1H), 5.01 (t, *J* = 9.6 Hz, 1H), 4.86 (d, *J* = 2.8 Hz, 1H), 4.81 (d, *J* = 9.6 Hz, 1H), 4.76 (d, *J* = 12.4 Hz, 1H), 4.54-4.59 (m, 2H), 4.17-4.29 (m, 1H), 4.05-4.14 (m, 2H), 3.78-3.90 (m, 1H), 2.33 (s, 3H), 1.88-2.01 (m, 18H), 1.70-1.80 (m, 1H), 1.40-1.50 (m, 1H); ^{13}C NMR ($CDCl_3$, 400MHz): δ 171.2, 170.8, 170.6, 170.4, 170.3, 169.4, 162.7, 143.7, 137.8, 129.8, 126.8, 121.8, 96.0, 88.3, 85.7, 74.7, 72.2, 68.4, 67.0, 64.6, 62.6, 61.9, 60.3, 53.9, 48.0, 36.5, 32.6, 22.7, 21.4, 20.8, 20.4, 14.1; HRMS (ESI) m/z $[M + Na]^+$ calcd for $C_{34}H_{45}N_5O_{16}SNa$ 834.2480, found 834.2484.

1-(2'-*N*-Acetyl-3',4',6'-tri-*O*-acetyl-α-D-glucopyranosyl)-4-(4'-(2'',3'',4'',6''-tetra-*O*-acetyl-α-D-glucopyranoside)-6'-*O*-acetyl-2',3'-dideoxy-α-D-allopyranosyloxymethyl)-1*H*-1,2,3-triazol (**4k**): Compound **4k** (gummy liquid, 44 mg, 80% yield) was prepared according to the general procedure (method A) for the synthesis of multivalent 3-tosylamino-2,3-dideoxyneoglycoconjugates from propargyl 3-tosylamino-2,3-dideoxysugar **2d** (36 mg, 0.05 mmol), benzyl azide **3g** (28 mg, 0.075 mmol), $CuSO_4$·$5H_2O$ solution (5 μL, 1.0 M), and

sodium ascorbate solution (5 μL, 1.0 M) in DMF (2 mL). The crude product was purified by column chromatography (hexane/ethyl acetate = 1:1); compound **4k** (gummy liquid, 45 mg, 82% yield) was also prepared according to the general procedure (method B) for the synthesis of multivalent 3-tosylamino-2,3-dideoxyneoglycoconjugates from propargyl 3-tosylamino-2,3-dideoxysugar **2d** (36 mg, 0.05 mmol), azide **3g** (28 mg, 0.075 mmol), $CuSO_4{\cdot}5H_2O$ solution (5 μL, 1.0 M), and sodium ascorbate solution (5 μL, 1.0 M) in DMF (2 mL) under microwave irradiation. The crude product was purified by column chromatography (hexane/ethyl acetate = 1:1); ^{1}H NMR ($CDCl_3$, 400MHz): δ 7.95 (s, 1H), 7.53(d, *J* = 8.4 Hz, 2H), 7.27 (d, *J* = 9.6 Hz, 2H), 6.40 (d, *J* = 8.4 Hz, 1H), 6.31 (d, *J* = 10.0 Hz, 1H), 5.95 (d, *J* = 10.0 Hz, 1H), 5.62 (d, *J* = 9.2 Hz, 1H), 5.43 (dd, *J* = 10.0, 9.2 Hz, 1H), 5.37 (d, *J* = 3.6 Hz, 1H), 5.19-5.28 (m, 2H), 5.06-5.13 (m, 1H), 4.86 (s, 1H), 4.76-4.82 (m, 2H), 4.58 (d, *J* = 12.0 Hz, 1H), 4.50 (dd, *J* = 12.0, 2.8 Hz, 1H), 4.07-4.28 (m, 8H), 3.90-3.97 (m, 1H), 3.78-3.88 (m, 1H), 3.74 (dd, *J* = 10.4, 3.6 Hz, 1H), 2.41 (s, 3H), 2.14 (s, 3H), 1.96-2.10 (m, 25H), 1.49-1.54 (m, 1H); ^{13}C NMR ($CDCl_3$, 400MHz): δ 171.0, 170.6, 170.5, 170.4, 170.3, 170.0, 169.5, 169.4, 169.3, 144.1, 143.7, 137.9, 130.0, 126.7, 121.4, 96.4, 88.4, 85.6, 75.0, 73.9, 72.2, 71.6, 69.9, 68.7, 68.4, 68.1, 65.7, 63.1, 61.8, 60.5, 60.4, 54.7, 54.1, 46.2, 31.3, 23.2, 22.8, 21.5, 21.1, 20.9, 20.7, 20.6, 20.5, 20.4; HRMS (ESI) m/z [M + Na]$^+$ calcd for $C_{46}H_{61}N_5O_{24}SNa$ 1122.3325, found 1122.3322.

1-(2',2',7',7'-Tetramethyltetrahydro-bis([1,3]dioxolo)pyranylmethyl)-4-(3''-*p*-toluenesulfonamido-4'',6''-di-*O*-acetyl-2'',3''-dideoxy-α-D-allo-pyranosyloxymethyl)-1*H*-1,2,3-triazol (**4l**): Compound **4l** (gummy liquid, 26 mg, 72% yield) was prepared according to the general procedure (method A) for the synthesis of multivalent 3-tosylamino-2,3-dideoxyneoglycoconjugates from propargyl 3-tosylamino-2,3-dideoxysugar **2a** (22 mg, 0.05 mmol), azide **3h** (22 mg, 0.075 mmol), $CuSO_4·5H_2O$ solution (5 μL, 1.0 M), and sodium ascorbate solution (5 μL, 1.0 M) in DMF (2 mL). The crude product was purified by column chromatography (hexane/ethyl acetate = 1:1); compound **4l** (gummy liquid, 28 mg, 78% yield) was also prepared according to the general procedure (method B) for the synthesis of multivalent 3-tosylamino-2,3-dideoxyneoglycoconjugates from propargyl 3-tosylamino-2,3-dideoxysugar **2a** (22 mg, 0.05 mmol), azide **3h** (22 mg, 0.075 mmol), $CuSO_4·5H_2O$ solution (5 μL, 1.0 M), and sodium ascorbate solution (5 μL, 1.0 M) in DMF (2 mL) under microwave irradiation. The crude product was purified by column chromatography (hexane/ethyl acetate = 1:1); ^{1}H NMR ($CDCl_3$, 400MHz): δ 7.76 (s, 1H), 7.69 (d, *J* = 8.0 Hz, 2H), 7.27 (d, *J* = 7.6 Hz, 2H), 5.95 (d, *J* = 8.8 Hz, 1H), 5.52 (d, *J* = 5.2 Hz, 1H), 4.93 (d, *J* = 3.6 Hz, 1H), 4.84 (d, *J* = 12.4 Hz, 1H), 4.63-4.69 (m, 4H), 4.48 (dd, *J* = 10.0, 8.8 Hz, 1H), 4.31-4.35 (m, 2H), 4.19-4.24 (m, 4H), 3.88-3.91 (m, 1H), 2.41 (s, 3H), 2.09 (s, 3H), 2.03 (s, 3H), 1.82 (dt, *J* = 14.8, 4.0 Hz, 1H), 1.51 (s, 3H), 1.49 (dd, *J* = 9.2, 2.4 Hz, 1H), 1.41 (s, 3H), 1.38 (s, 3H), 1.29 (s, 3H); ^{13}C NMR ($CDCl_3$,

400MHz): δ 170.7, 170.4, 143.3, 137.9, 129.7, 126.9, 124.2, 110.0, 109.1, 96.2, 96.0, 71.2, 70.8, 70.3, 67.2, 67.0, 64.6, 62.7, 60.6, 50.7, 47.9, 32.8, 26.0, 24.9, 24.4, 21.5, 21.1, 20.9, 20.8, 14.2; HRMS (ESI) m/z $[M + Na]^+$ calcd for $C_{32}H_{44}N_4O_{13}SNa$ 747.2523, found 747.2531.

1,3-Bis{4'-(3''-*p*-toluenesulfonamido-4'',6''-di-*O*-acetyl-2'',3''-dideoxy-α-D-allopyranosyloxymethyl)-1'*H*-1',2',3'-triazol-1'-methyl}-benzene (**6a**): Compound **6a** (gummy liquid, 44 mg, 83% yield) was prepared according to the general procedure (method A) for the synthesis of multivalent 3-tosylamino-2,3-dideoxyneoglycoconjugates from propargyl 3-tosylamino-2,3-dideoxysugar **2a** (44 mg, 0.1 mmol), diazide **5a** (10 mg, 0.05 mmol), $CuSO_4{\cdot}5H_2O$ solution (5 μL, 1.0 M), and sodium ascorbate solution (5 μL, 1.0 M) in DMF (2 mL). The crude product was purified by column chromatography (hexane/ethyl acetate = 1:1); compound **6a** (gummy liquid, 46 mg, 86% yield) was also prepared according to the general procedure (method B) for the synthesis of multivalent 3-tosylamino-2,3-dideoxyneoglycoconjugates from propargyl 3-tosylamino-2,3-dideoxysugar **2a** (44 mg, 0.1 mmol), diazide **5a** (10 mg, 0.05 mmol), $CuSO_4{\cdot}5H_2O$ solution (5 μL, 1.0 M), and sodium ascorbate solution (5 μL, 1.0 M) in DMF (2 mL) under microwave irradiation. The crude product was purified by column chromatography (hexane/ethyl acetate = 1:1); ^1H NMR ($CDCl_3$, 400MHz): δ 7.59-7.62 (m, 6H), 7.40-7.44 (m, 1H), 7.23-7.30 (m, 7H) , 5.99 (d, *J* = 8.8 Hz, 2H), 5.55 (s, 4H), 4.98 (d, *J* = 3.2 Hz, 2H), 4.82 (d, *J* = 12.0

Hz, 2H), 4.57-4.65 (m, 4H), 4.26-4.36 (m, 2H), 4.12-4.17 (m, 4H), 3.86-3.89 (m, 2H), 2.40 (s, 6H), 2.04 (s, 6H), 1.98 (s, 6H), 1.83 (dt, *J* = 14.8, 4.0 Hz, 2H), 1.56 (d, *J* = 2.8 Hz, 2H); ^{13}C NMR ($CDCl_3$, 400MHz): δ 170.7, 170.2, 143.4, 137.7, 135.8, 129.8, 128.5, 127.6, 126.9, 122.7, 96.6, 66.9, 64.7, 62.6, 60.8, 60.4, 54.3, 48.0, 32.9, 21.5, 21.0, 20.8, 20.7, 14.2; HRMS (ESI) m/z $[M + Na]^+$ calcd for $C_{48}H_{58}N_8O_{16}S_2Na$ 1089.3310, found 1089.3312.

1,3-Bis{4’-(3’’-*p*-toluenesulfonamido-4’’-*O*-acetyl-2’’,3’’,6’’-trideoxy-α-L-ribohexopyranosyloxymethyl)-1’*H*-1’,2’,3’-triazol-1’-methyl}benzene (**6b**): Compound **6b** (gummy liquid, 28 mg, 61% yield) was prepared according to the general procedure (method A) for the synthesis of multivalent 3-tosylamino-2,3-dideoxyneoglycoconjugates from propargyl 3-tosylamino-2,3-dideoxysugar **2c** (38 mg, 0.1 mmol), diazide **5a** (10 mg, 0.05 mmol), $CuSO_4{\cdot}5H_2O$ solution (5 μL, 1.0 M), and sodium ascorbate solution (5 μL, 1.0 M) in DMF (2 mL). The crude product was purified by column chromatography (hexane/ethyl acetate = 1:1); compound **6b** (gummy liquid, 30 mg, 65% yield) was also prepared according to the general procedure (method B) for the synthesis of multivalent 3-tosylamino-2,3-dideoxyneoglycoconjugates from propargyl 3-tosylamino-2,3-dideoxysugar **2c** (38 mg, 0.1 mmol), diazide **5a** (10 mg, 0.05 mmol), $CuSO_4{\cdot}5H_2O$ solution (5 μL, 1.0 M), and sodium ascorbate solution (5 μL, 1.0 M) in DMF (2 mL) under microwave irradiation. The crude product was purified by column chromatography (hexane/ethyl acetate = 1:1); ^1H NMR

(CDCl$_3$, 400MHz): δ 7.62 (d, *J* = 8.0 Hz, 4H), 7.54 (s, 2H), 7.41-7.44 (m, 2H), 7.23-7.34 (m, 6H), 5.97 (d, *J* = 8.8 Hz, 2H), 5.58 (s, 4H), 4.88 (d, *J* = 2.4 Hz, 2H), 4.82 (d, *J* = 12.0 Hz, 2H), 4.59 (d, *J* =12.4 Hz, 2H), 4.32-4.61 (m, 2H), 3.99-4.07 (m, 2H), 3.80-3.83 (m, 2H), 2.40 (s, 6H), 2.01 (s, 6H), 1.76-1.81 (m, 2H), 1.51 (d, *J* = 14.4 Hz, 2H), 1.18 (d, *J* = 6.0 Hz, 6H); ^{13}C NMR (CDCl$_3$, 400MHz): δ 170.6, 144.4, 143.2, 137.9, 136.7, 135.2, 129.8, 129.7, 129.6, 128.6, 128.0, 127.8, 126.9, 122.4, 96.2, 72.5, 62.3, 60.6, 54.3, 54.0, 48.0, 33.1, 21.5, 21.0, 17.4, 14.2; HRMS (ESI) m/z $[M+H]^+$ calcd for $C_{44}H_{55}N_8O_{12}S_2$ 951.3381, found 951.3361.

2,4,6-Tris{2-[4'-(3''-*p*-toluenesulfonamido-4'',6''-di-*O*-acetyl-2'',3''-dideoxy-α-D-allopyranosyloxymethyl)-1'*H*-1',2',3'-triazol-1'-yl]ethoxy}-1,3,5-triazine (**6c**): Compound **6c** (gummy liquid, 54 mg, 66% yield) was prepared according to the general procedure (method A) for the synthesis of multivalent 3-tosylamino-2,3-dideoxyneoglycoconjugates from propargyl 3-tosylamino-2,3-dideoxysugar **2a** (66 mg, 0.15 mmol), triazide **5b** (17 mg, 0.05 mmol), $CuSO_4 \cdot 5H_2O$ solution (5 μL, 1.0 M), and sodium ascorbate solution (5 μL, 1.0 M) in DMF (2 mL). The crude product was purified by column chromatography (hexane/ethyl acetate = 1:1); compound **6c** (gummy liquid, 56 mg, 68% yield) was also prepared according to the general procedure (method B) for the synthesis of multivalent 3-tosylamino-2,3-dideoxyneoglycoconjugates from propargyl 3-tosylamino-2,3-dideoxysugar **2a** (66 mg, 0.15 mmol), triazide **5b** (17 mg,

0.05 mmol), $CuSO_4 \cdot 5H_2O$ solution (5 μL, 1.0 M), and sodium ascorbate solution (5 μL, 1.0 M) in DMF (2 mL) under microwave irradiation. The crude product was purified by column chromatography (hexane/ethyl acetate = 1:1); ^{1}H NMR ($CDCl_3$, 400MHz): δ 7.78 (s, 3H), 7.66 (d, *J* = 8.4 Hz, 6H), 7.29 (d, *J*= 8.8 Hz, 6H), 5.93 (d, *J* = 9.2 Hz, 3H), 4.98 (d, *J* = 3.2 Hz, 3H), 4.83-4.87 (m, 6H), 4.62-4.65 (m, 3H), 4.56 (t, *J* = 4.8 Hz, 6H), 4.31 (dd, *J* = 12.0, 4.8 Hz, 3H), 4.18-4.20 (m, 6H), 3.86-3.89 (m, 3H), 3.65 (t, *J* = 4.8 Hz, 6H), 2.41 (s, 9H), 2.08 (s, 9H), 2.01 (s, 9H), 1.84 (dt, *J* = 14.8, 4.0 Hz, 3H), 1.56 (dd, *J* = 12.0, 2.8 Hz, 3H); ^{13}C NMR ($CDCl_3$, 400MHz): δ 173.1, 171.8, 171.6, 170.8, 170.4, 143.4, 137.7, 129.8, 126.9, 96.4, 67.4, 66.9, 64.6, 62.6, 60.6, 49.4, 48.9, 47.9, 32.8, 21.5, 20.9; HRMS (ESI) m/z $[M + Na]^+$ calcd for $C_{69}H_{87}N_{15}O_{27}S_3Na$ 1676.4956, found 1676.4979.

References

1. Shull, B. K.; Wu, Z.; Koreeda, M. *J. Carbohydr. Chem.* **1996**, *15*, 955.
2. Tang, K.-C.; Mariuzza, R.; Coward, J. K. *J. Med. Chem.* **1981**, *24*, 1277.
3. Liu, C.; Coward, J. K. *J. Med. Chem.* **1991**, *34*, 2094.
4. Maier, M. A.; Yannopoulos, C. G.; Mohamed, N.; Roland, A.; Fritz, H.; Mohan, V.; Just, G.; Manoharan, M. *Bioconjugate Chem.* **2003,** *14,* 18.
5. Hou, D.-R.; Kuan, T.-C.; Li, Y.-K.; Lee, R.; Huang, K.-W. *Tetrahedron*, **2010**, *66*, 9415-9420.
6. Alvarez, S. G.; Alvarez, M. T. *Synthesis* **1997**, 413.

7. Perez-Balderas, F.; Ortega-Munoz, M.; Morales-Sanfrutos, J.; Hernandez-Mateo, F.; Calvo-Flores, F. G.; Calvo-Asín, J. A.; Isac-García, J.; Santoyo-Gonzalez, F. *Org. Lett.* **2003**, *5*, 1951.
8. Theocharis, A. B.; Alexandrou, N. E.; Terzis, A. *J. Heterocycl. Chem.* **1990**, *27* 1741.
9. Wuytswinkel, G. V.; Verheyde, B.; Compernolle, F.; Toppet, S.; Dehaen, W. *J. Chem. Soc. Perkin I* **2000**, *9*, 1337.
10. Ding, F. Q.; William, R.; Cai, S. T.; Ma, J.; Liu, X.-W. *J. Org. Chem.* **2012***,* *77,* 5245.

NMR spectra for all compounds.

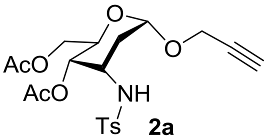

2a

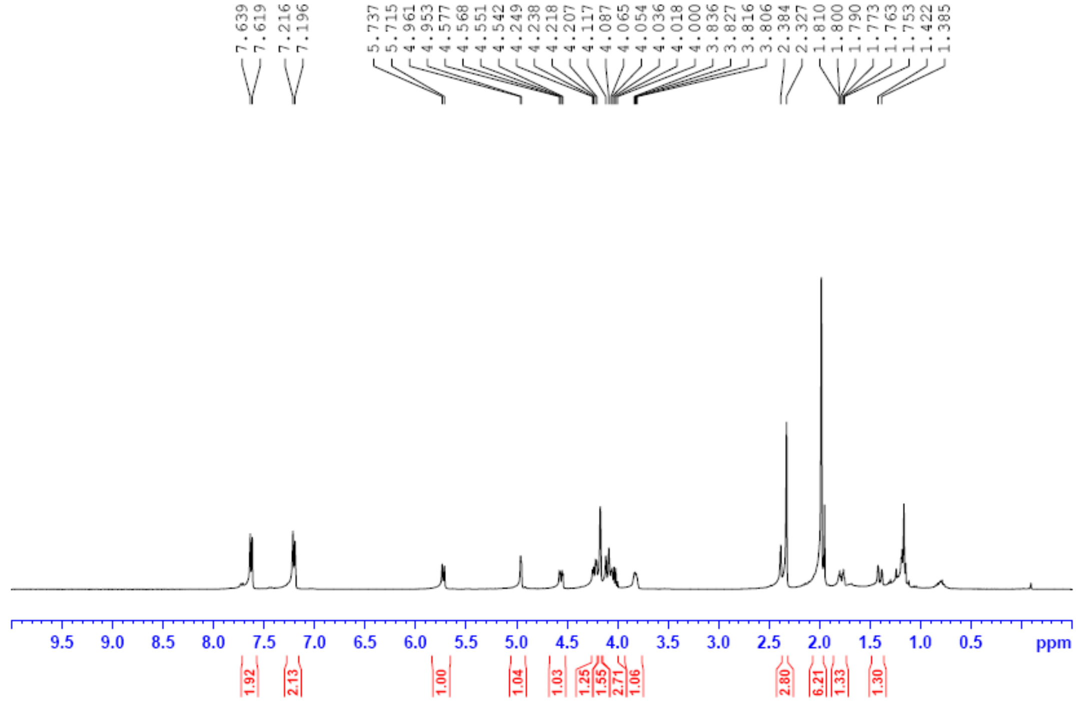

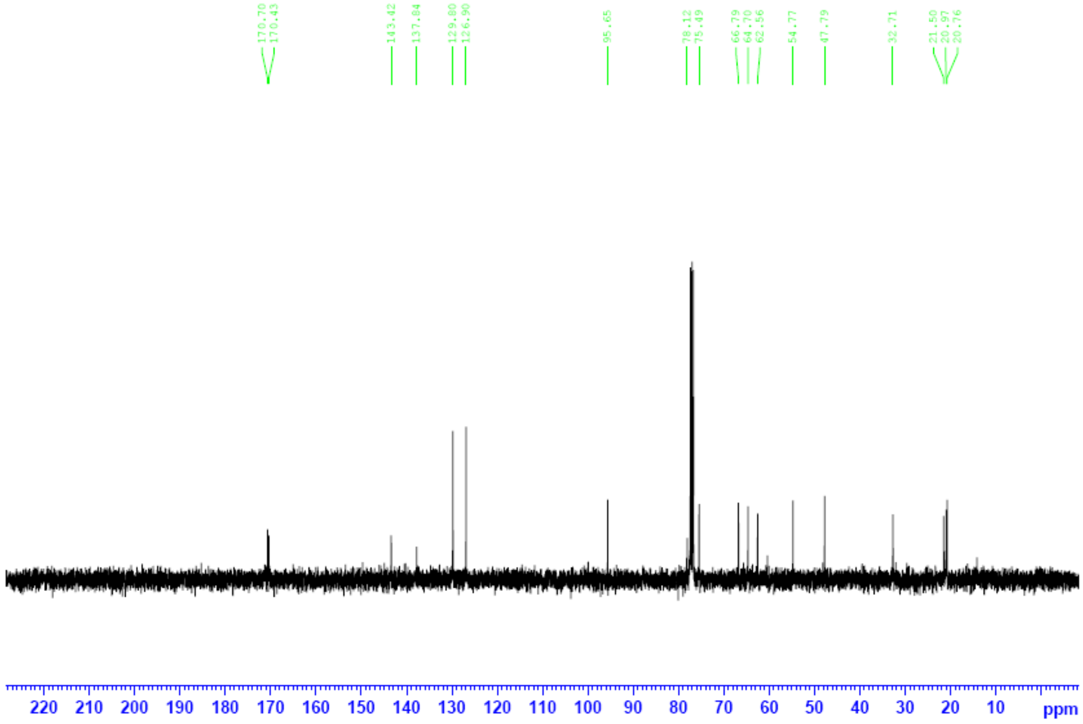

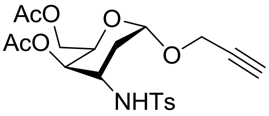

2b

D2-142-1p,1H NMR, JUL, BBFO1 400HZ

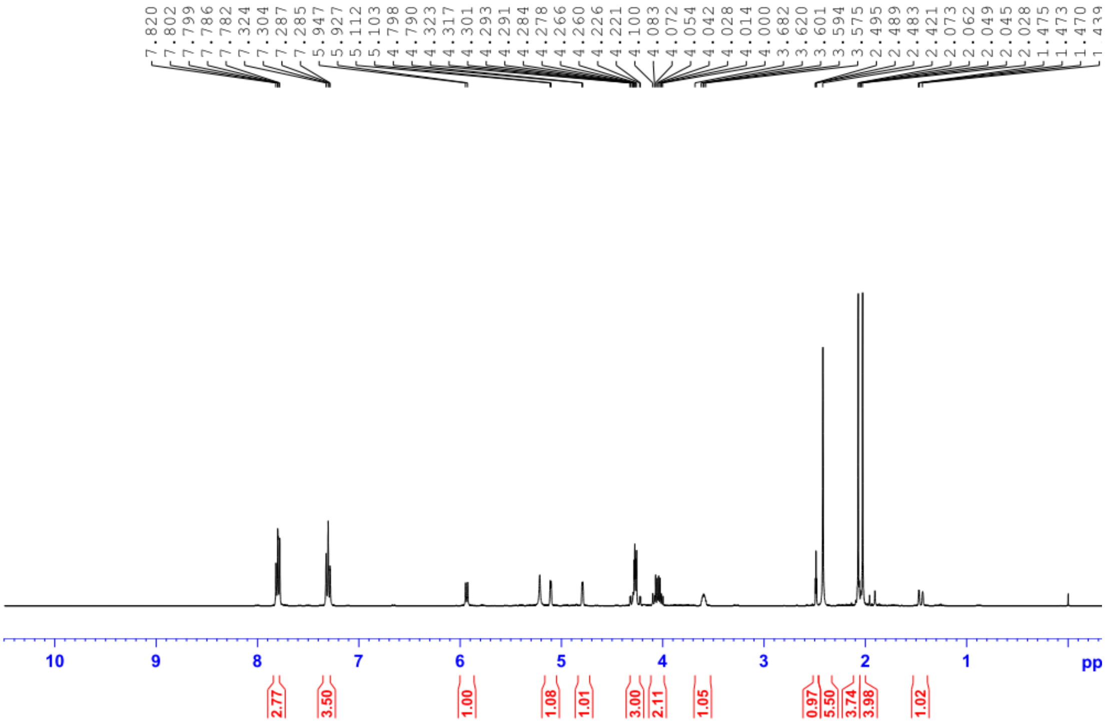

D2-142-1p,13C NMR, JUL, BBFO1 400HZ

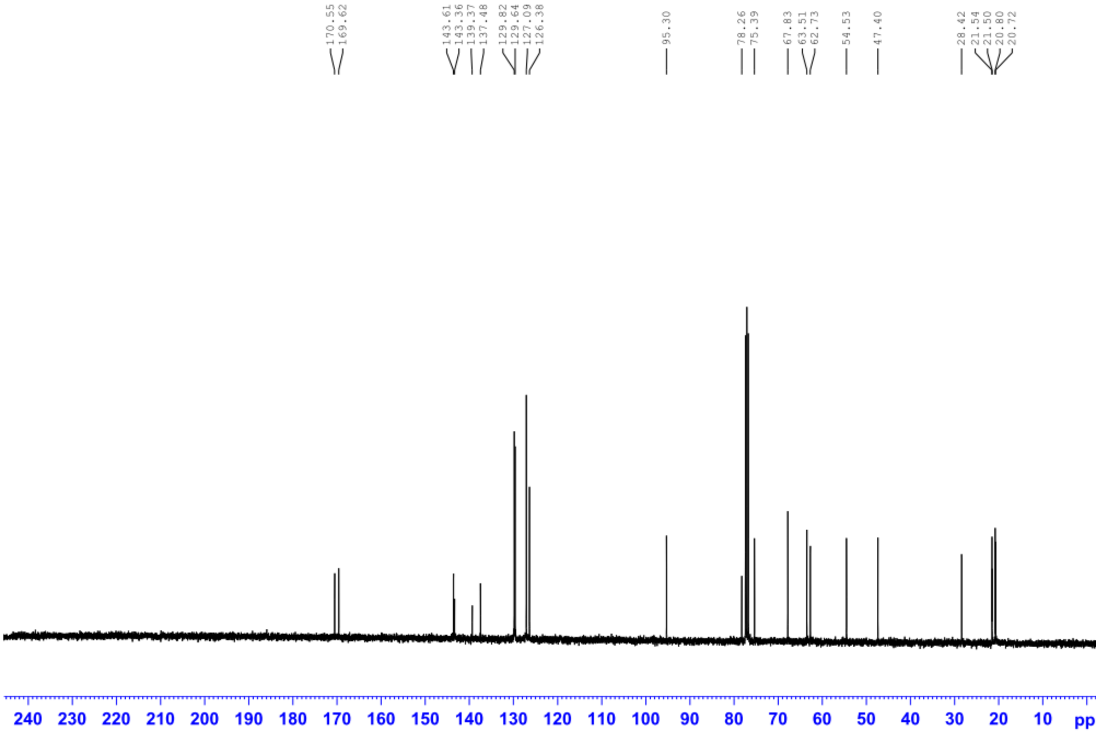

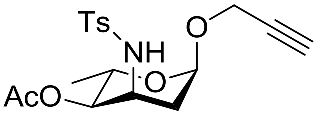
Ts
NH
O
O
AcO

2c

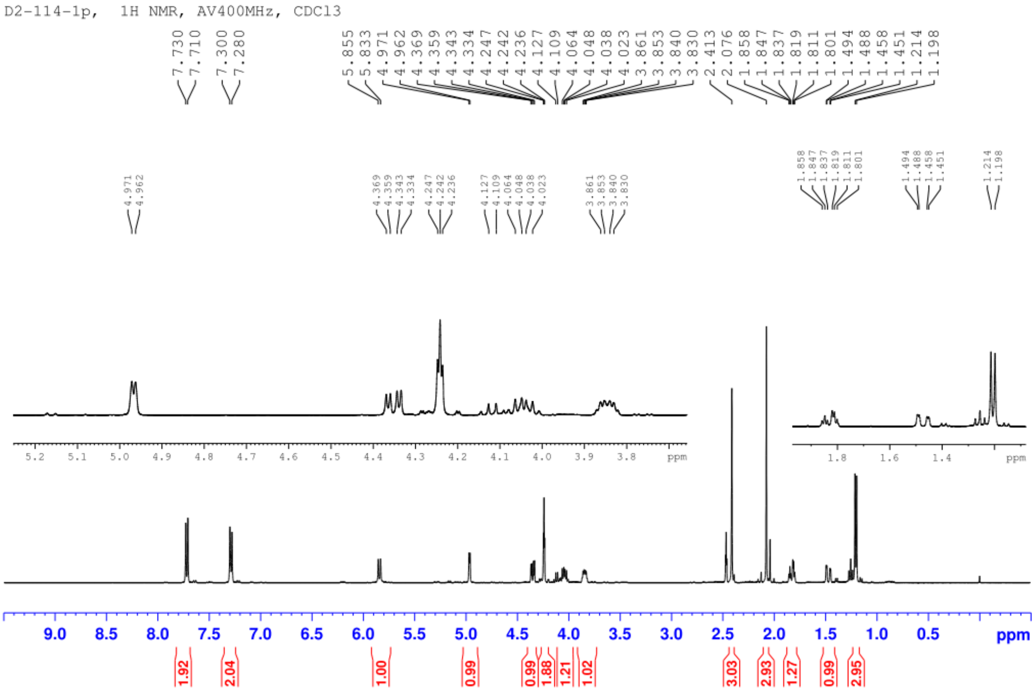
D2-114-1p, 1H NMR, AV400MHz, CDCl3
ppm

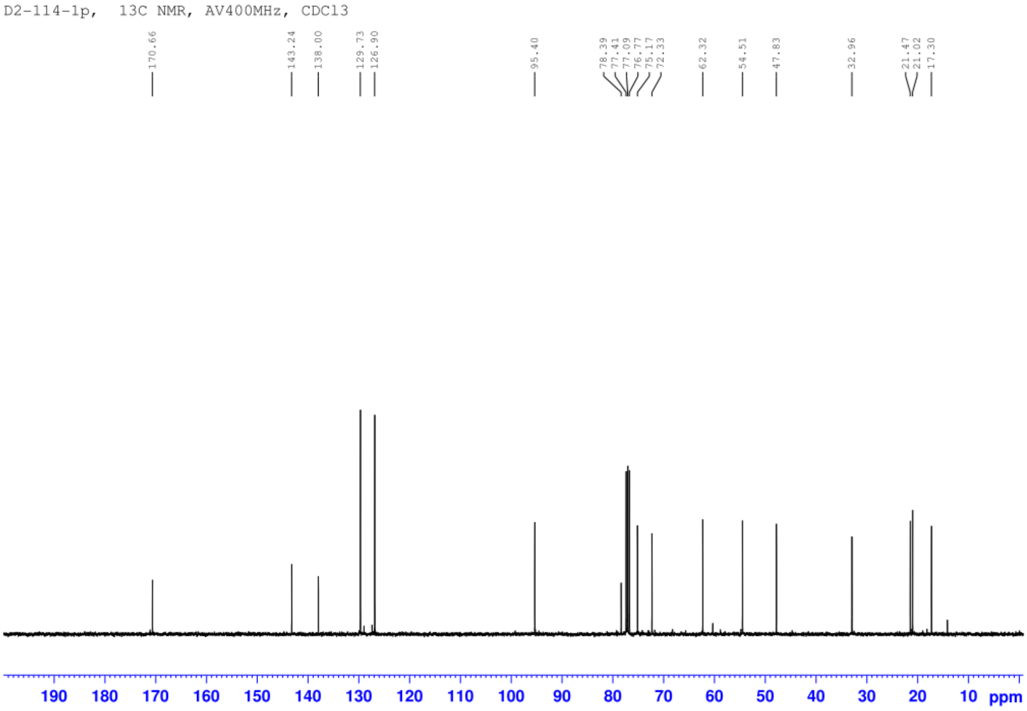
D2-114-1p, 13C NMR, AV400MHz, CDCl3
ppm

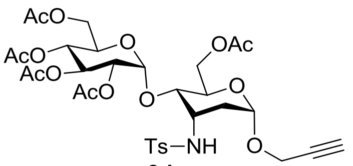

2d

D2-121-1p, 1H NMR, AV400MHz, CDCl3

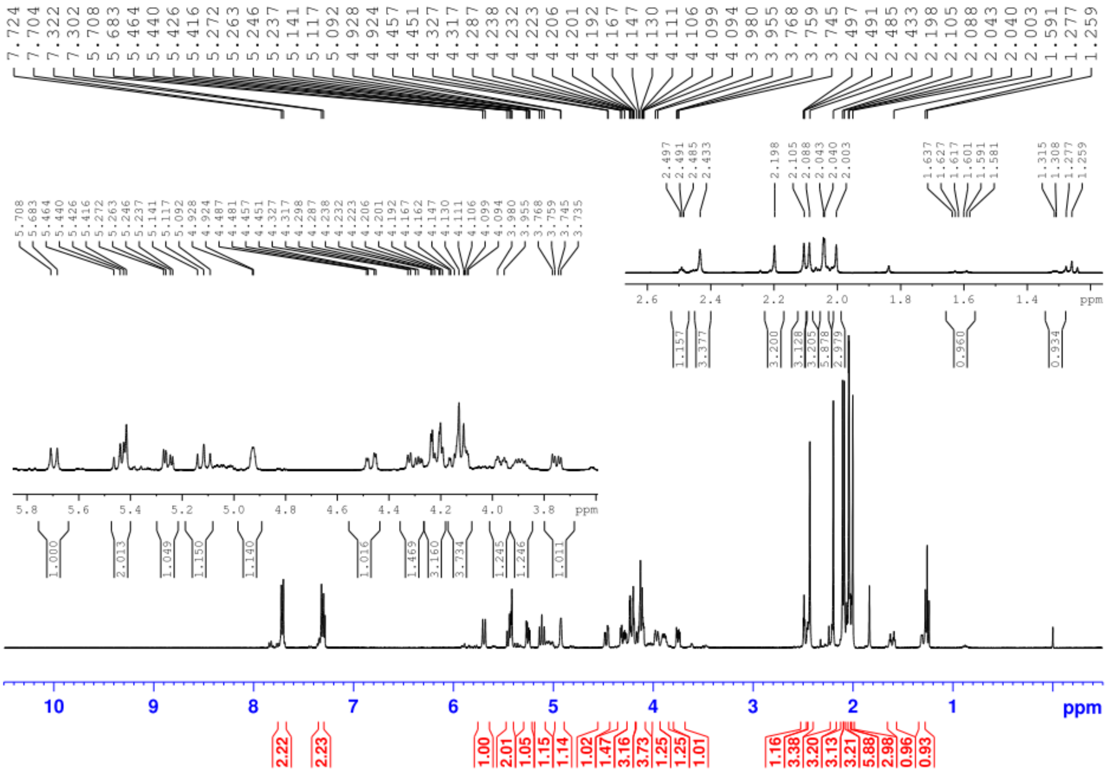

D2-121-1p, 13C NMR, AV400MHz, CDCl3

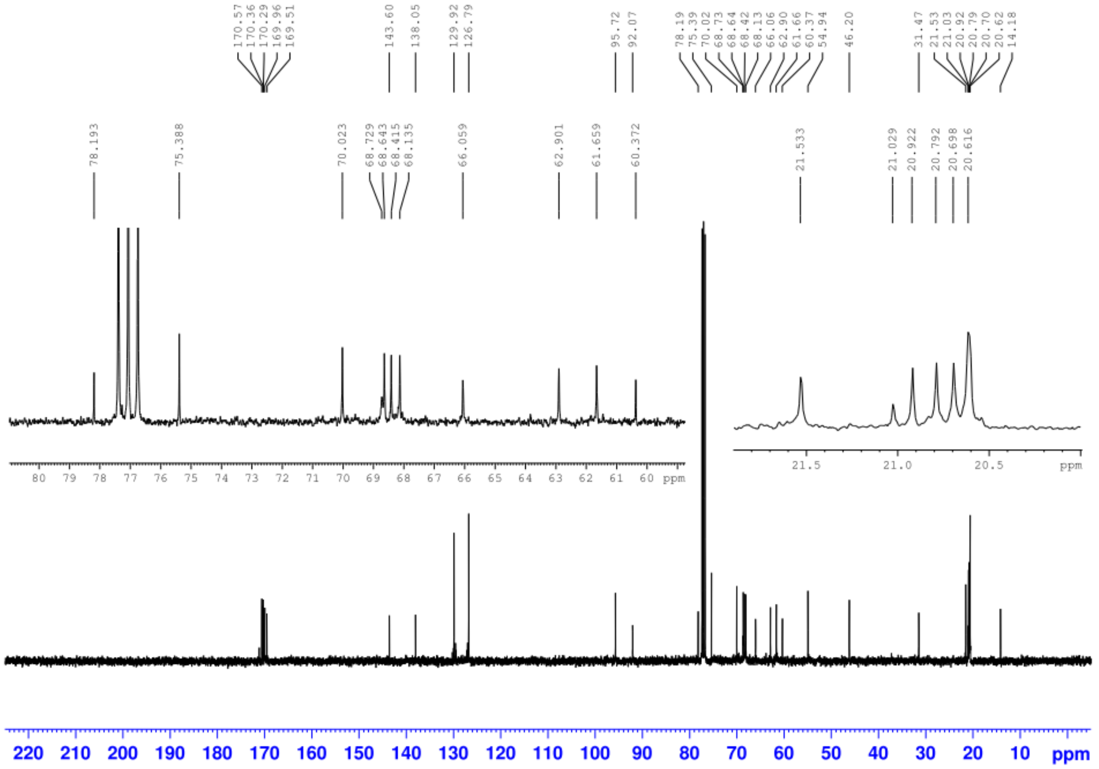

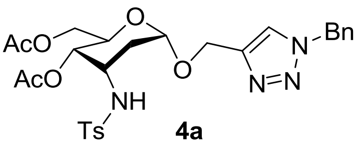

4a

D2-112-2p, 1H NMR, AV400MHz, CDCl3

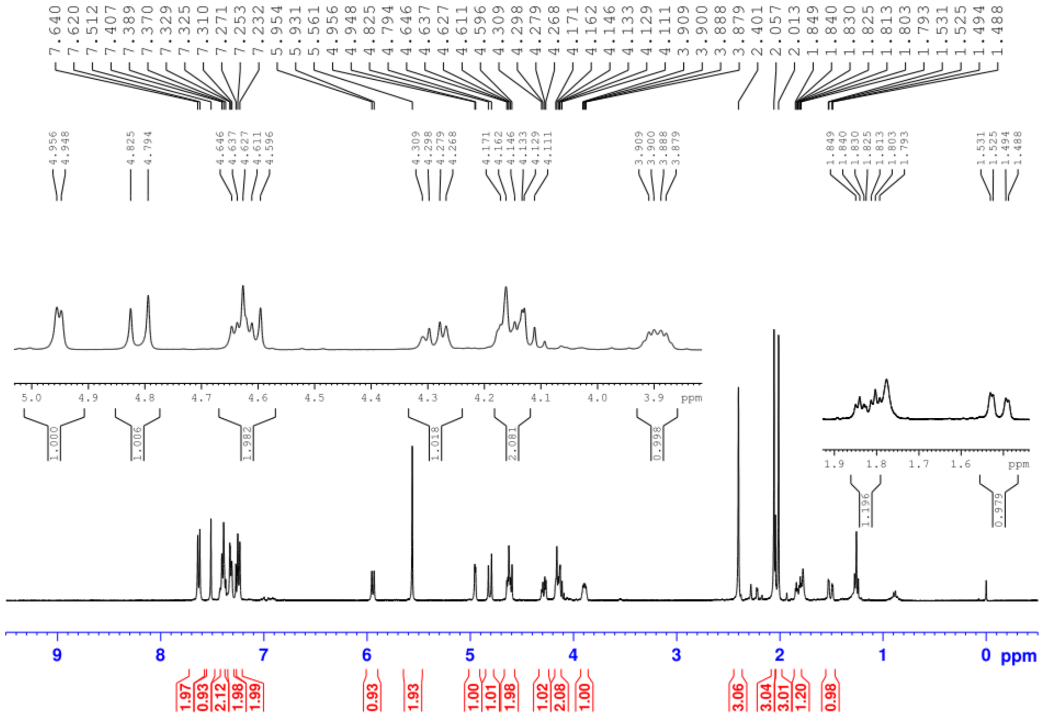

D2-112-2p, 13C NMR, AV400MHz, CDCl3

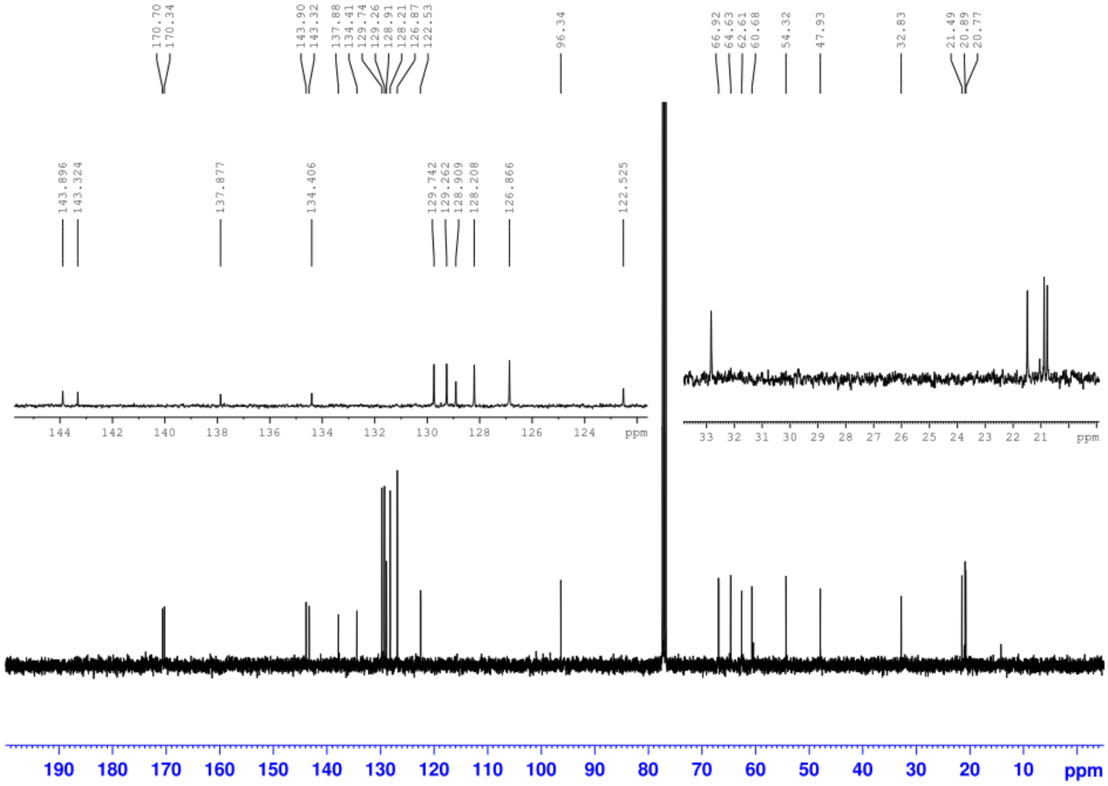

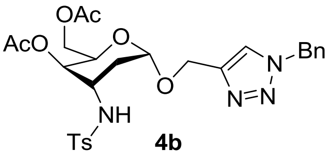

4b

D2-150-1P,1H, BBFO1,400 Hz, CDCl3

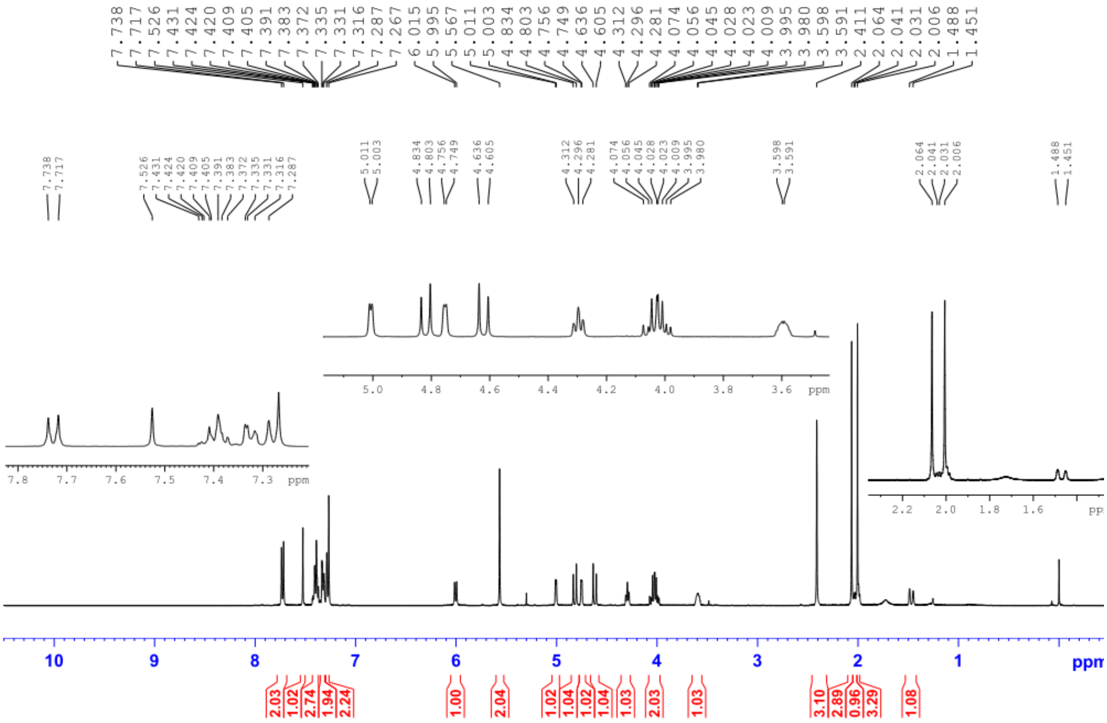

D2-150-1P,13C, BBFO1,400 Hz, CDCl3

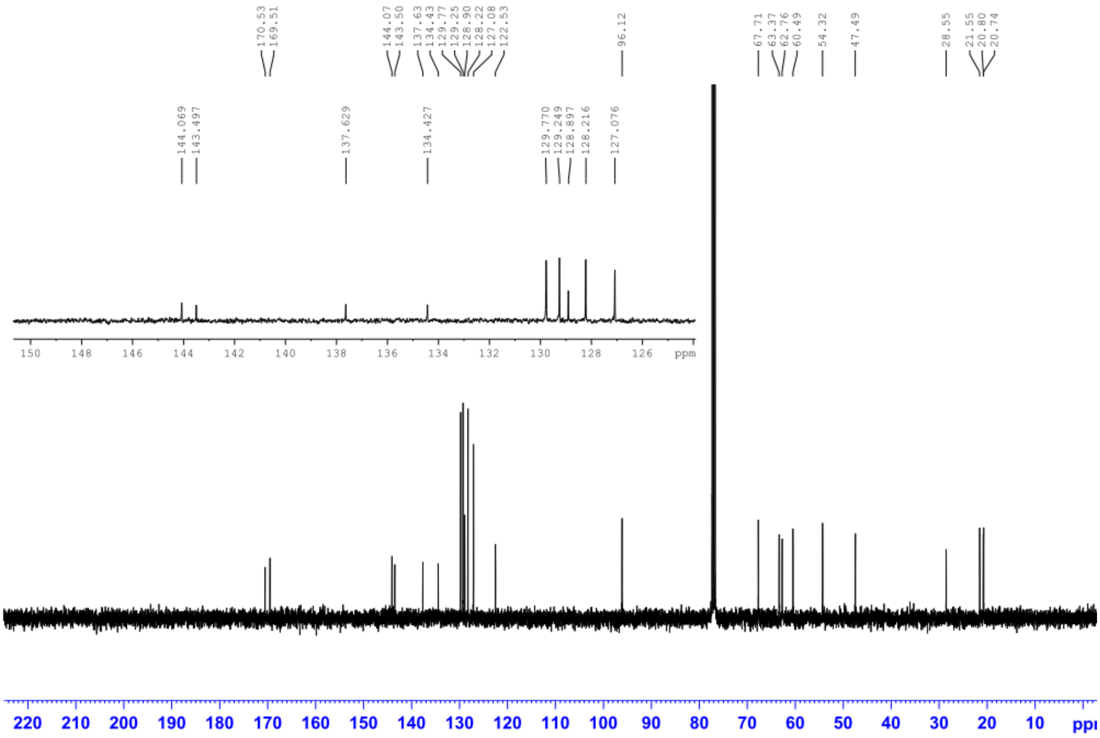

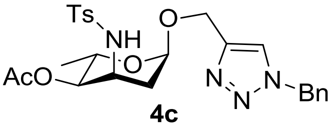

4c

D2-123-1P,1H, BBFO1,400 Hz, CDCl3

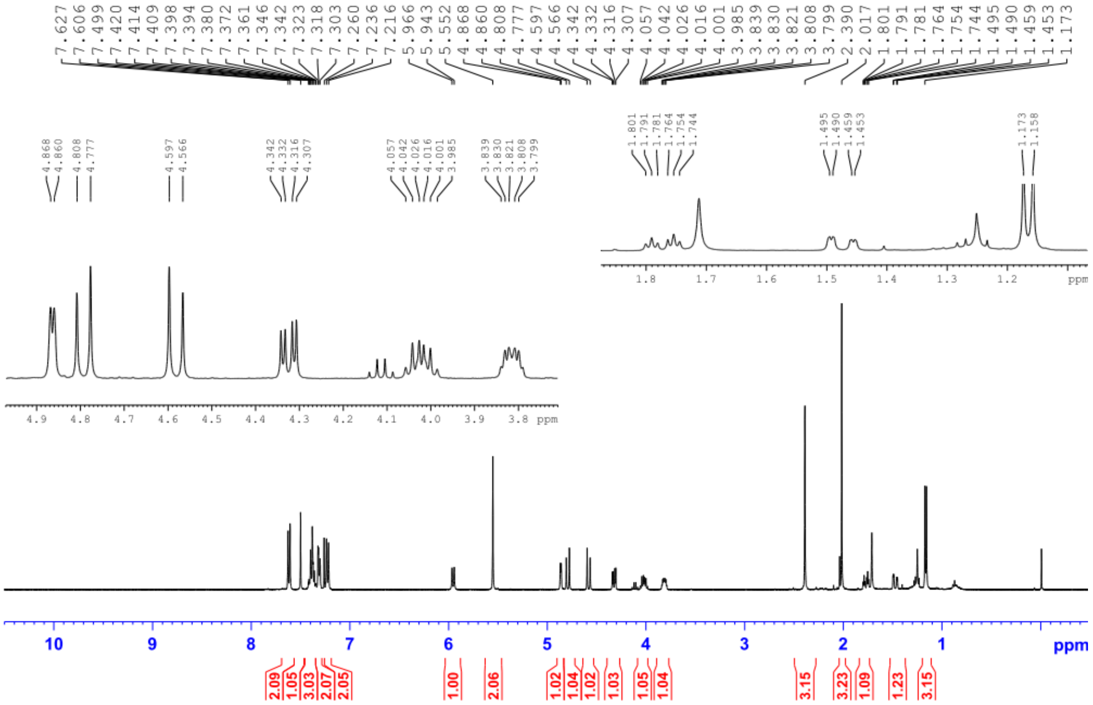

D2-123-1P,13C, BBFO1,400 Hz, CDCl3

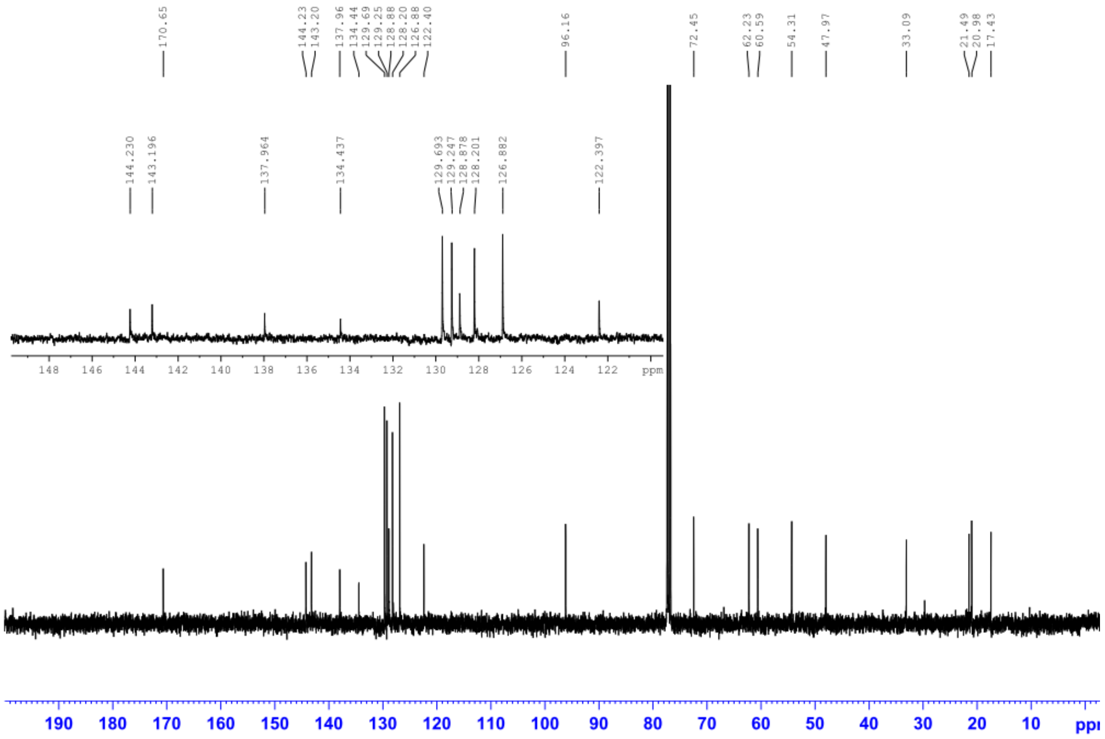

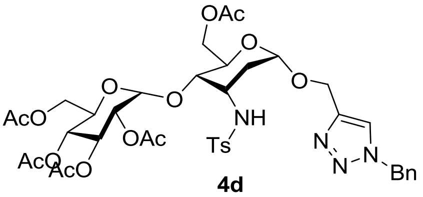
OAc
O
O
AcO
O
O
OAc
AcO
AcO
NH
Ts
O
N
N
N
Bn

4d

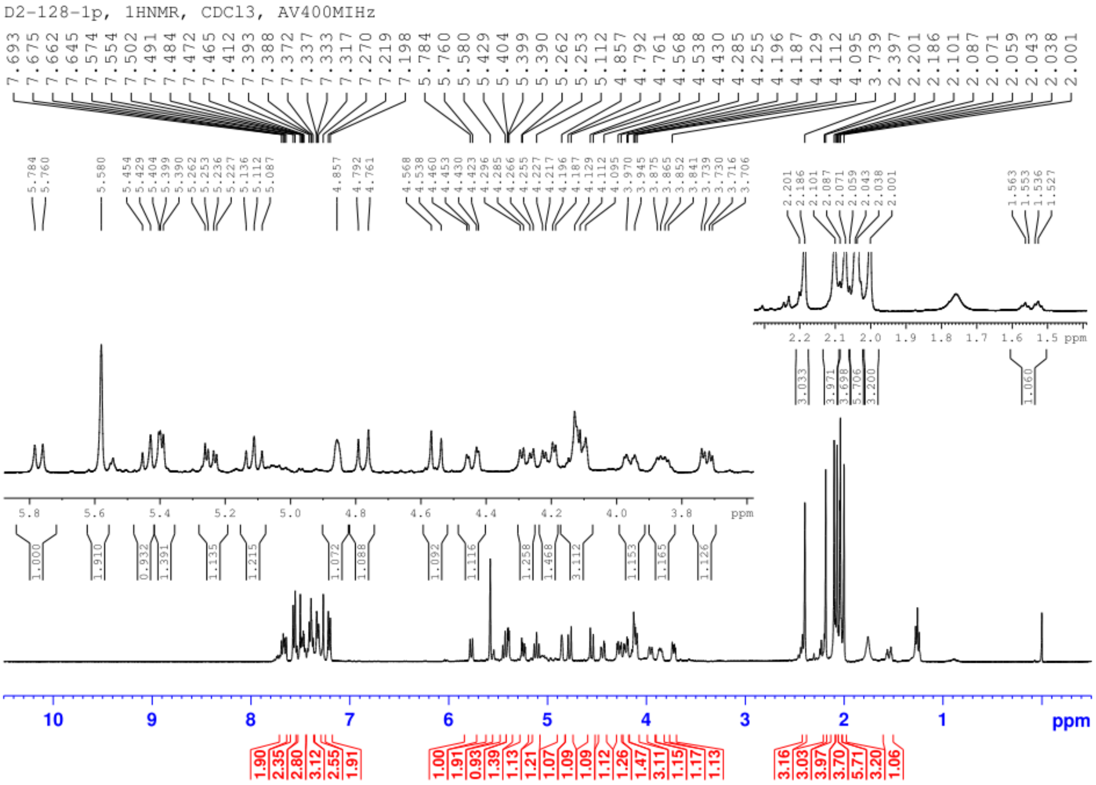
D2-128-1p, 1HNMR, CDCl3, AV400MIHz
7.693 7.675 7.662 7.645 7.574 7.554 7.502 7.491 7.484 7.472 7.465 7.412 7.393 7.388 7.372 7.337 7.333 7.317 7.270 7.219 7.198 5.784 5.760 5.580 5.429 5.404 5.399 5.390 5.262 5.253 5.112 4.857 4.792 4.761 4.568 4.538 4.430 4.285 4.255 4.196 4.187 4.129 4.112 4.095 3.739 2.397 2.201 2.186 2.101 2.087 2.071 2.059 2.043 2.038 2.001
ppm

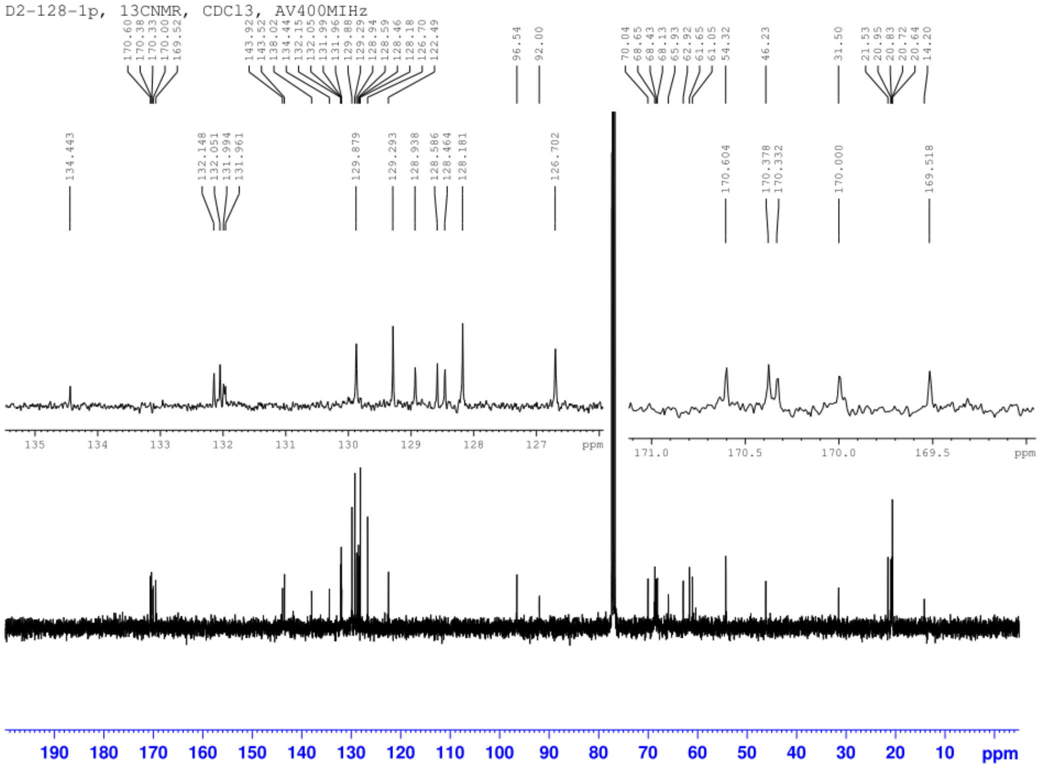
D2-128-1p, 13CNMR, CDCl3, AV400MIHz
170.60 170.38 170.33 170.00 169.52 143.92 143.52 138.02 134.44 132.15 132.05 131.99 131.96 129.88 129.29 128.94 128.59 128.46 128.18 126.70 122.49 96.54 92.00 70.04 68.65 68.43 68.13 65.93 62.92 61.65 61.05 54.32 46.23 31.50 21.53 20.95 20.83 20.72 20.64 14.20
ppm

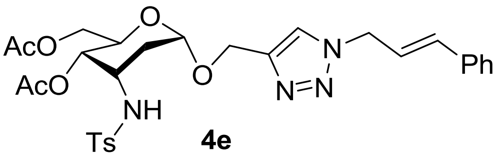

4e

D2-147-1p,1H NMR, JUL, BBFO1 400HZ

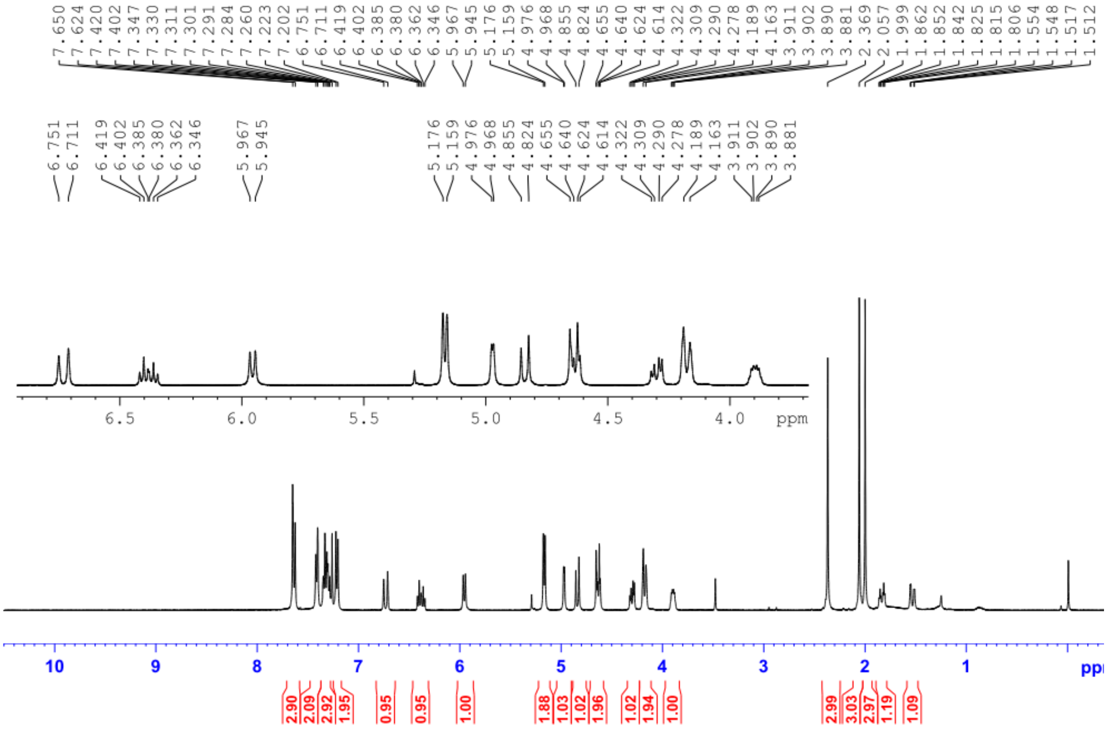

D2-147-1p,13C NMR, JUL, BBFO1 400HZ

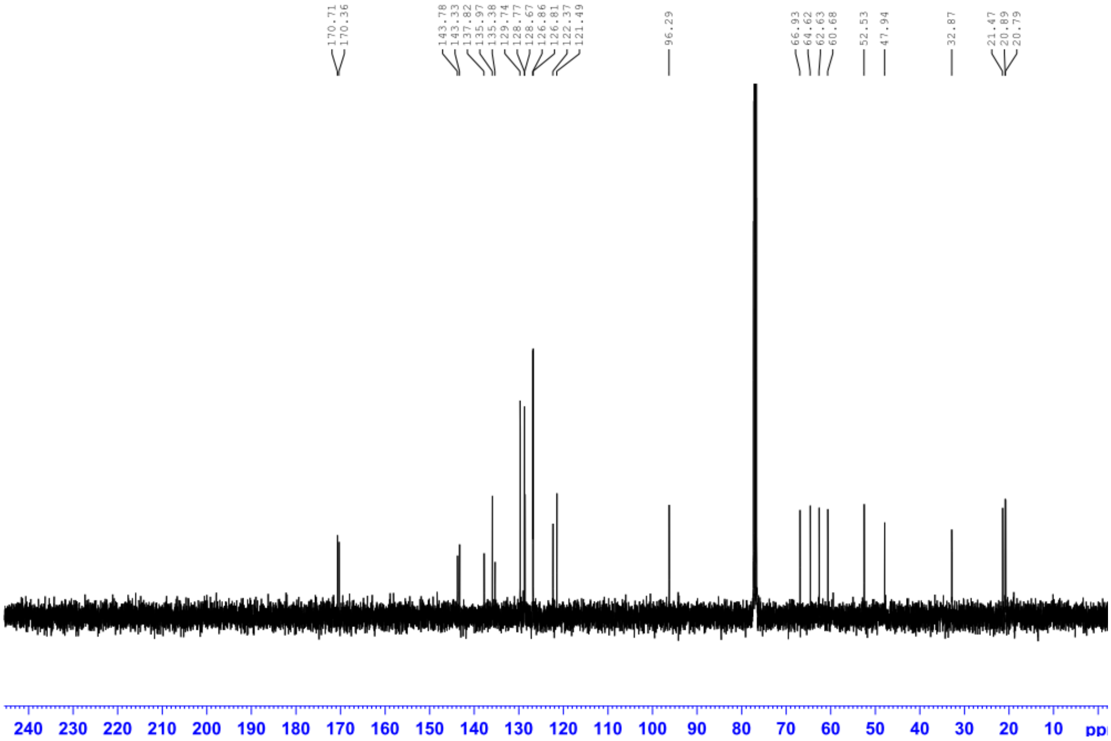

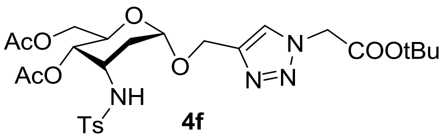

4f

D2-136-1p, 1HNMR, CDCl3, AV400MIHz

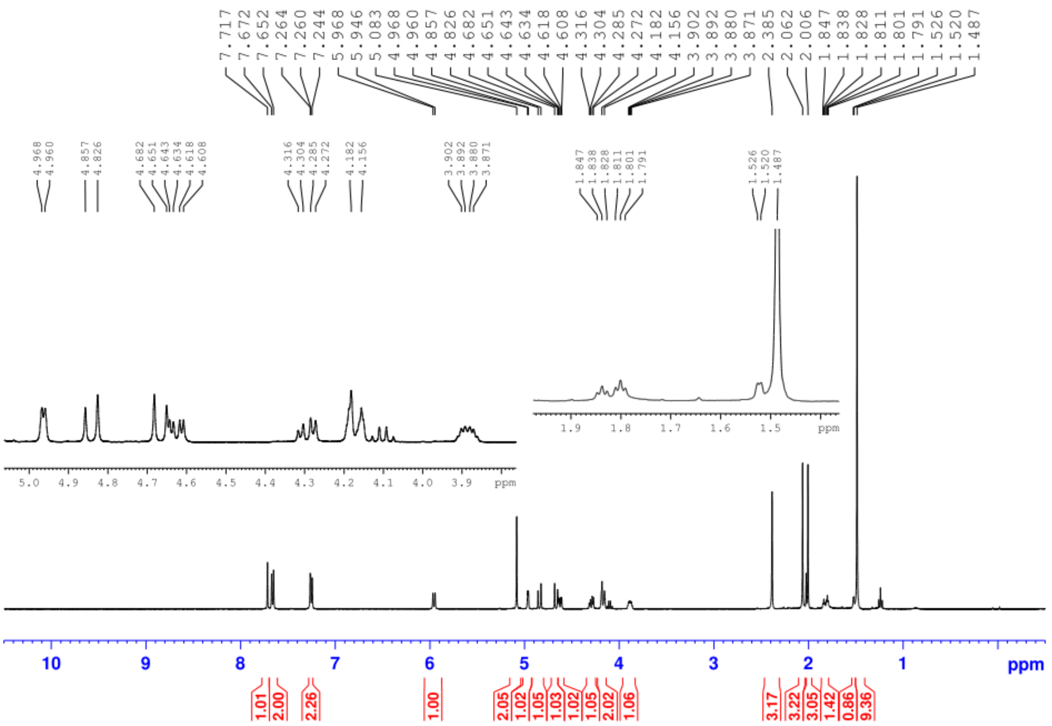

D2-136-1p, 13CNMR, CDCl3, AV400MIHz

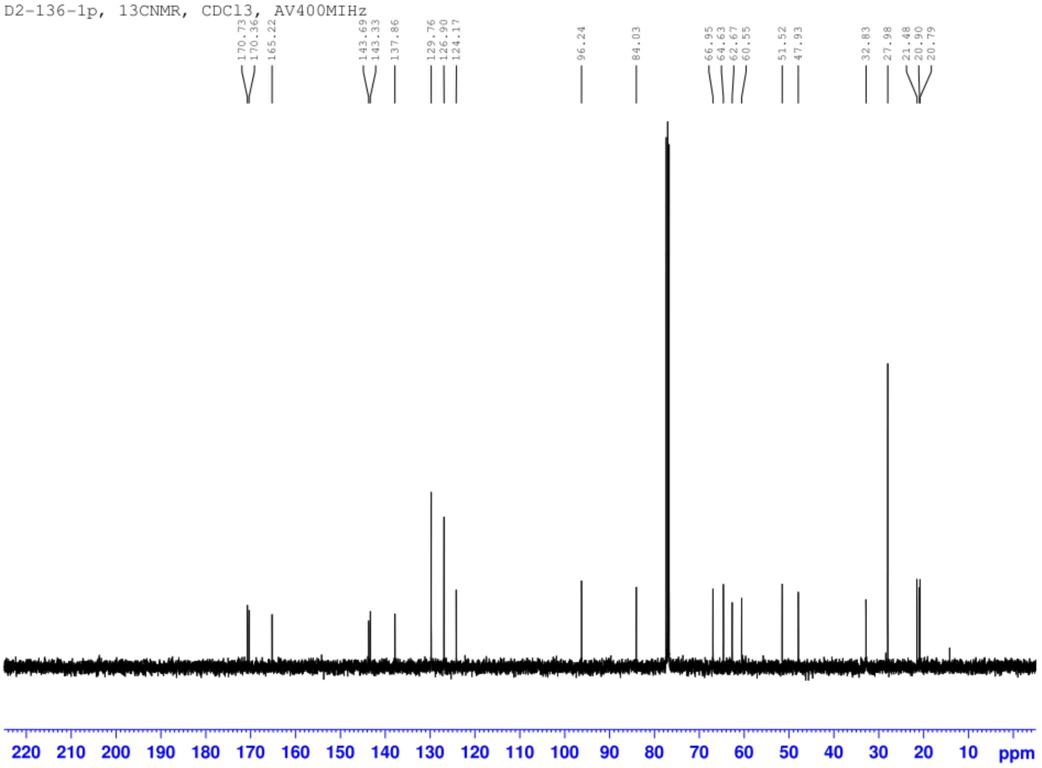

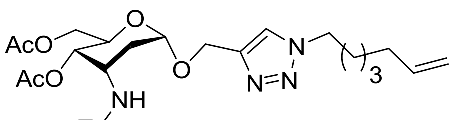
AcO
AcO
O
O
NH
Ts
N
N=N
3

4g

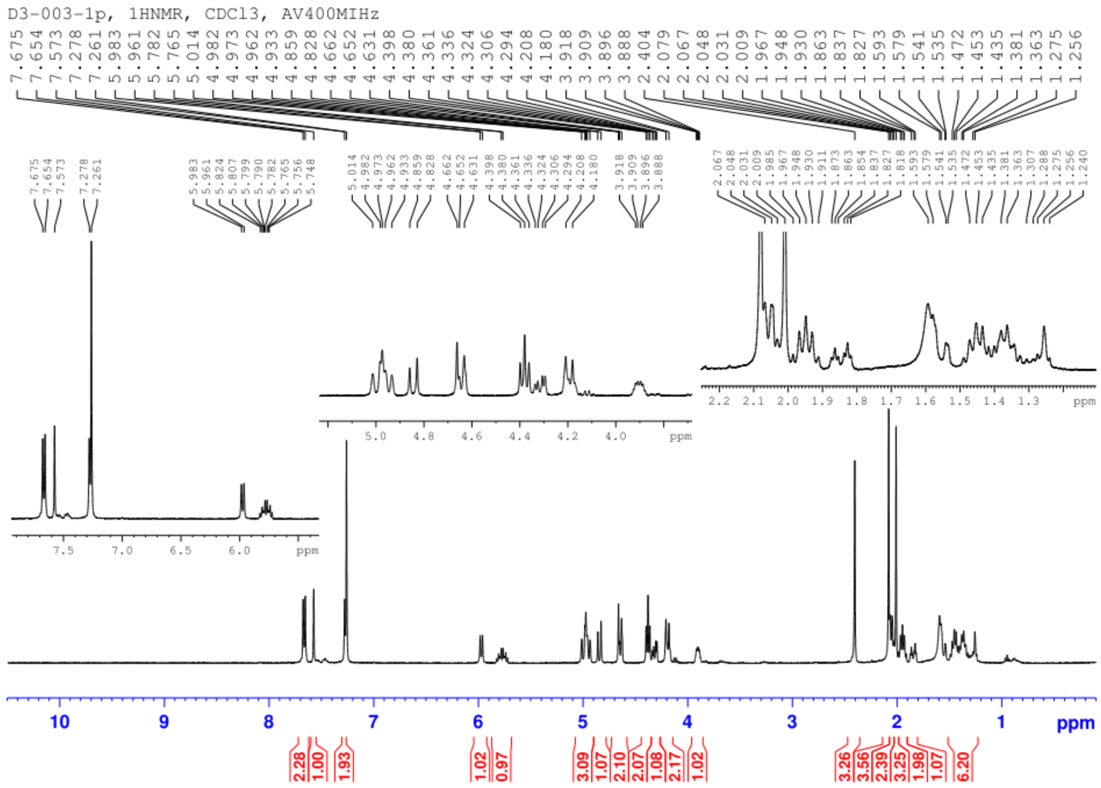
D3-003-1p, 1HNMR, CDCl3, AV400MIHz
ppm

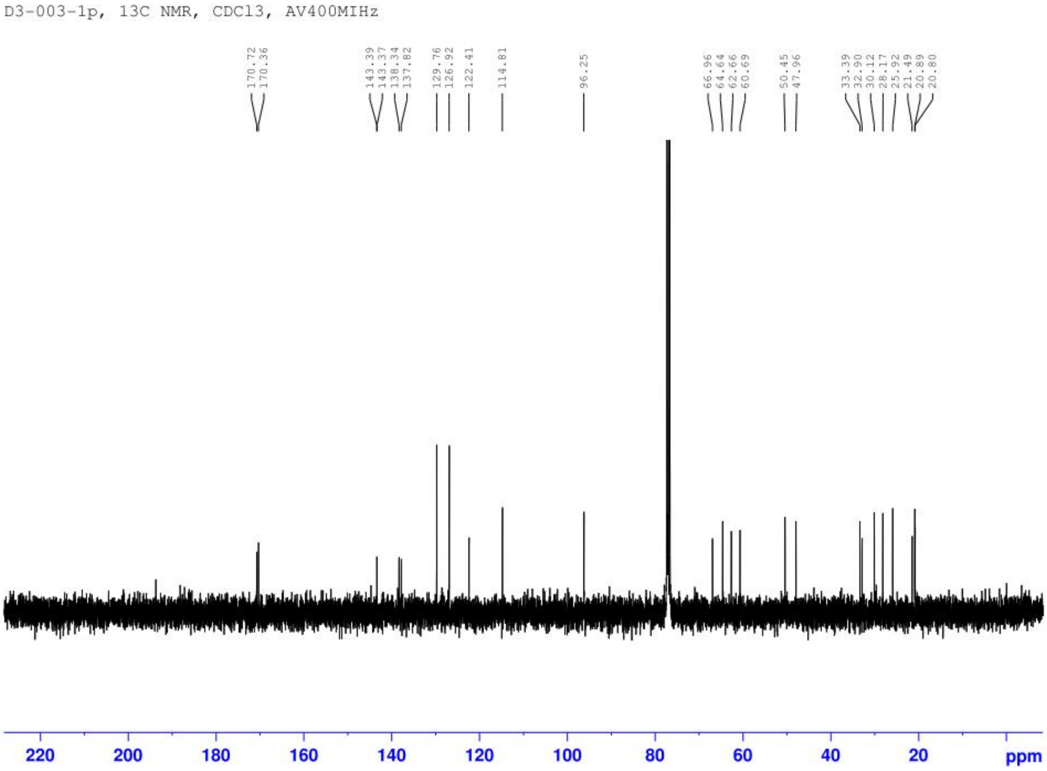
D3-003-1p, 13C NMR, CDCl3, AV400MIHz
ppm

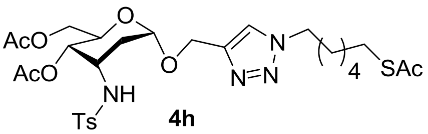
AcO
AcO
O
O
NH
Ts
N
N=N
4
SAc

4h

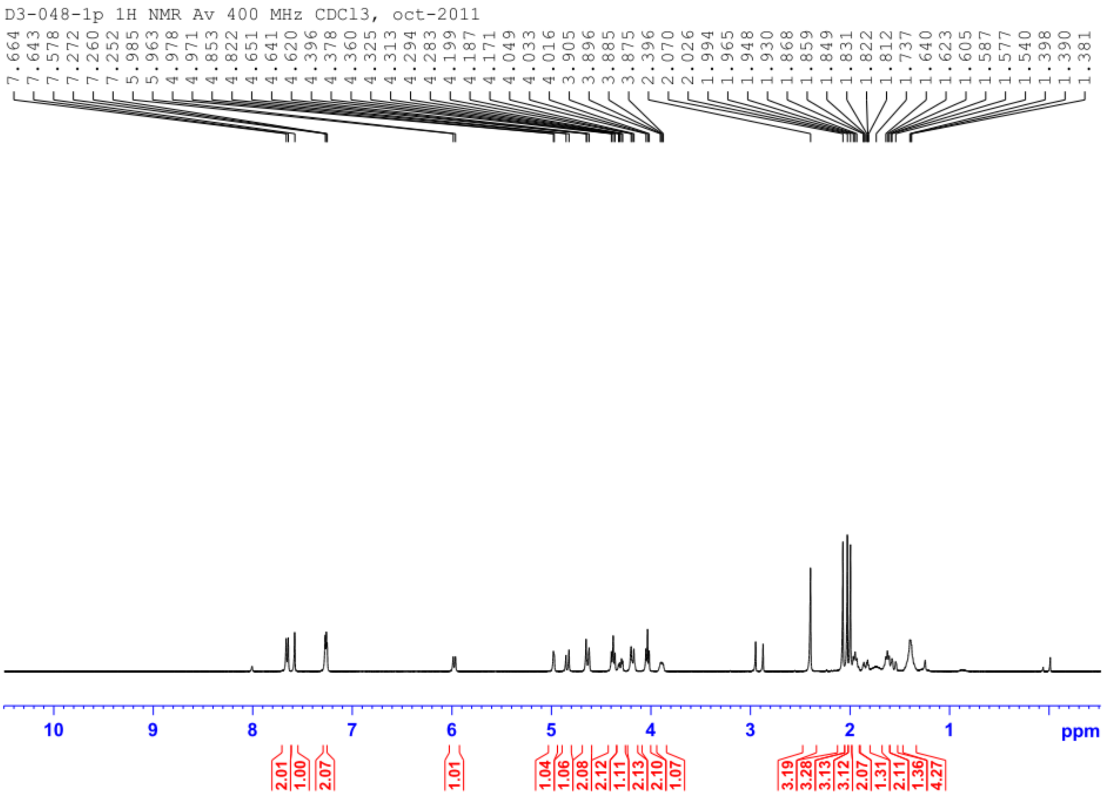
D3-048-1p 1H NMR Av 400 MHz CDCl3, oct-2011
7.664 7.643 7.578 7.272 7.260 7.252 5.985 5.963 4.978 4.971 4.853 4.822 4.651 4.641 4.620 4.396 4.378 4.360 4.325 4.313 4.294 4.283 4.199 4.187 4.171 4.049 4.033 4.016 3.905 3.896 3.885 3.875 2.396 2.070 2.026 1.994 1.965 1.948 1.930 1.868 1.859 1.849 1.831 1.822 1.812 1.737 1.640 1.623 1.605 1.587 1.577 1.540 1.398 1.390 1.381
10 9 8 7 6 5 4 3 2 1 ppm
2.01 1.00 2.07 1.01 1.04 1.06 2.08 2.12 1.11 2.13 2.10 1.07 3.19 3.28 3.13 3.12 2.07 1.31 2.11 1.36 4.27

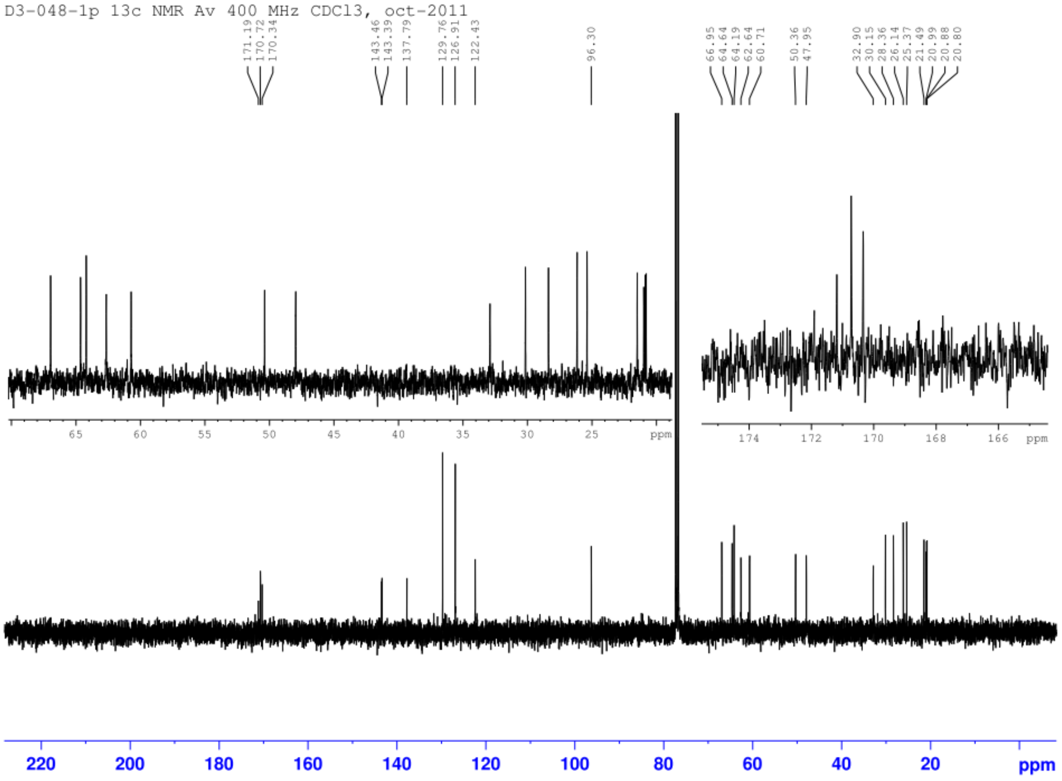
D3-048-1p 13c NMR Av 400 MHz CDCl3, oct-2011
171.19 170.72 170.34 143.46 143.39 137.79 129.76 126.91 122.43 96.30 66.95 64.64 64.19 62.64 60.71 50.36 47.95 32.90 30.15 28.36 26.14 25.37 21.49 20.99 20.88 20.80
65 60 55 50 45 40 35 30 25 ppm
174 172 170 168 166 ppm
220 200 180 160 140 120 100 80 60 40 20 ppm

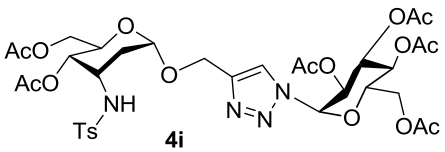
AcO
AcO
NH
Ts
O
O
N
N=N
AcO
OAc
OAc
OAc
O

4i

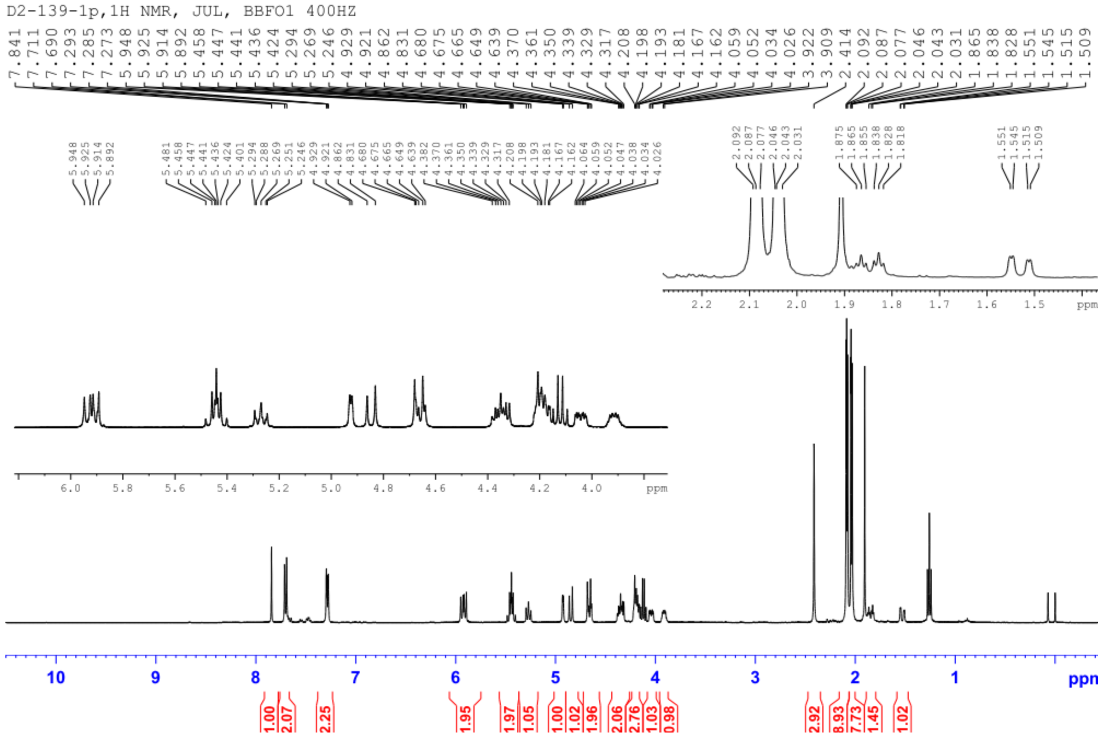
D2-139-1p,1H NMR, JUL, BBFO1 400HZ
7.841 7.711 7.690 7.293 7.285 7.273 5.948 5.925 5.914 5.892 5.458 5.447 5.441 5.436 5.424 5.294 5.269 5.246 4.929 4.921 4.862 4.831 4.680 4.675 4.665 4.649 4.639 4.370 4.361 4.350 4.339 4.329 4.317 4.208 4.198 4.193 4.181 4.167 4.162 4.059 4.052 4.034 4.026 3.922 3.909 2.414 2.092 2.087 2.077 2.046 2.043 2.031 1.865 1.838 1.828 1.551 1.545 1.515 1.509
10 9 8 7 6 5 4 3 2 1 ppm
1.00 2.07 2.25 1.95 1.97 1.05 1.00 1.02 1.96 2.06 2.76 1.03 0.98 2.92 8.93 7.73 1.45 1.02

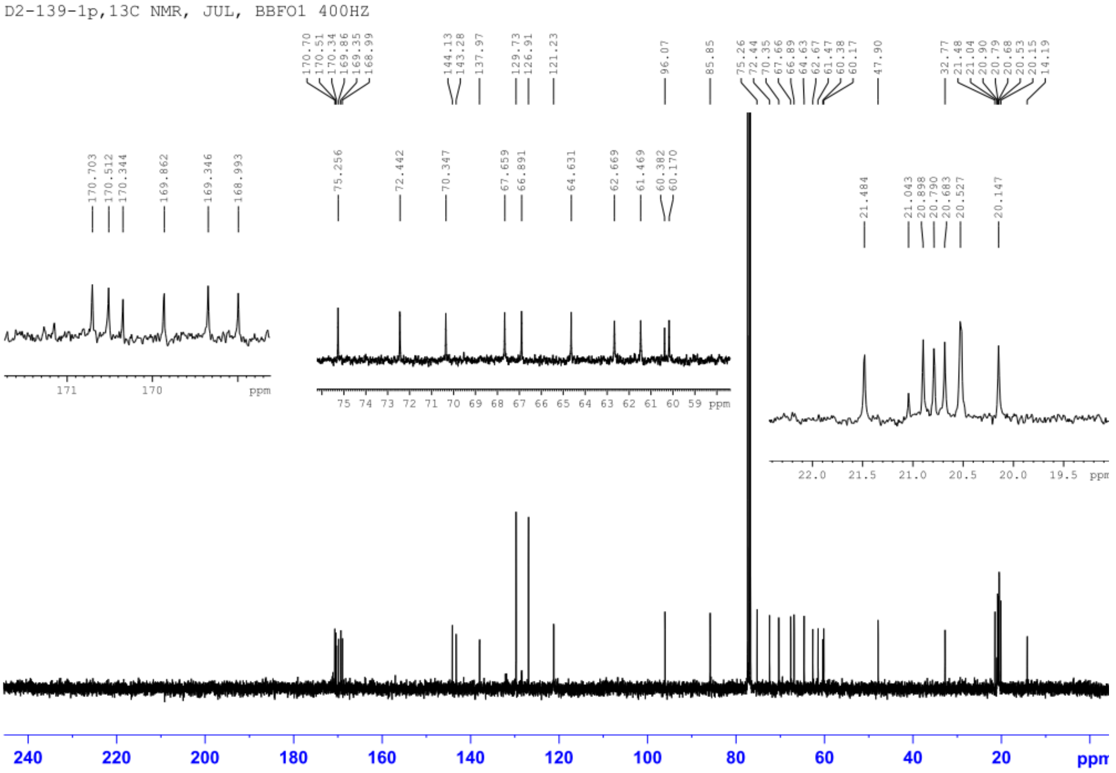
D2-139-1p,13C NMR, JUL, BBFO1 400HZ
170.70 170.51 170.34 169.86 169.35 168.99 144.13 143.28 137.97 129.73 126.91 121.23 96.07 85.85 75.26 72.44 70.35 67.66 66.89 64.63 62.67 61.47 60.38 60.17 47.90 32.77 21.48 21.04 20.90 20.79 20.68 20.53 20.15 14.19
240 220 200 180 160 140 120 100 80 60 40 20 ppm

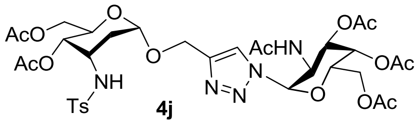
AcO
AcO
NH
Ts
O
O
N
N
N
AcHN
OAc
OAc
OAc
O

4j

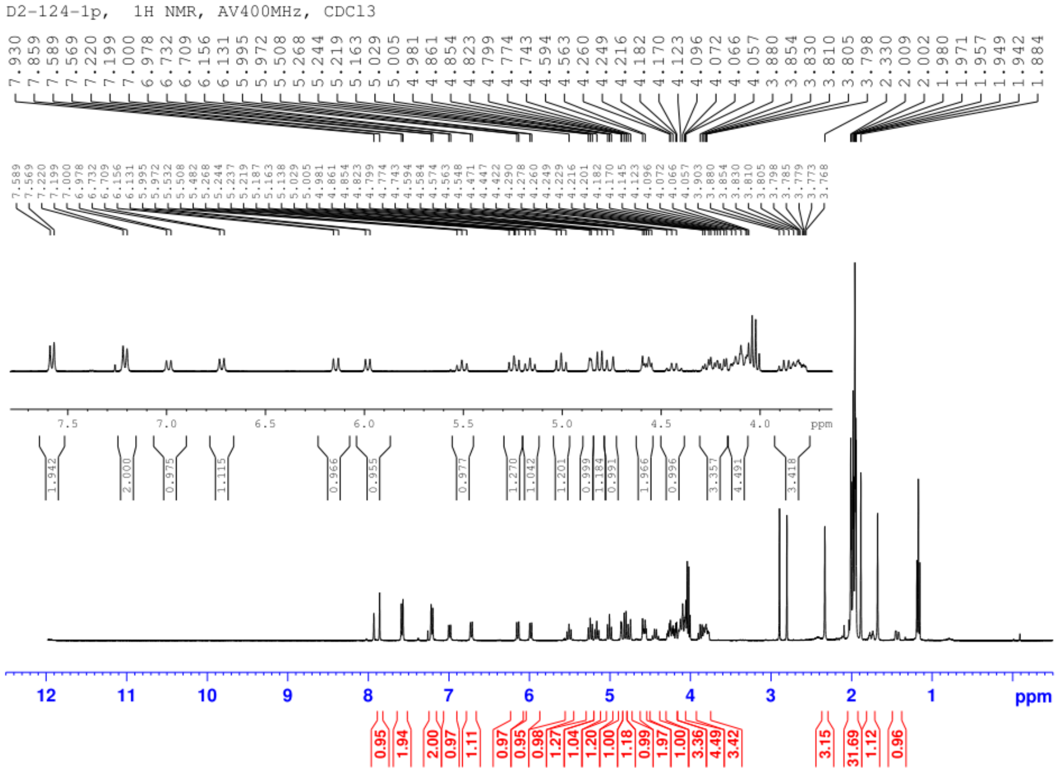
D2-124-1p, 1H NMR, AV400MHz, CDCl3

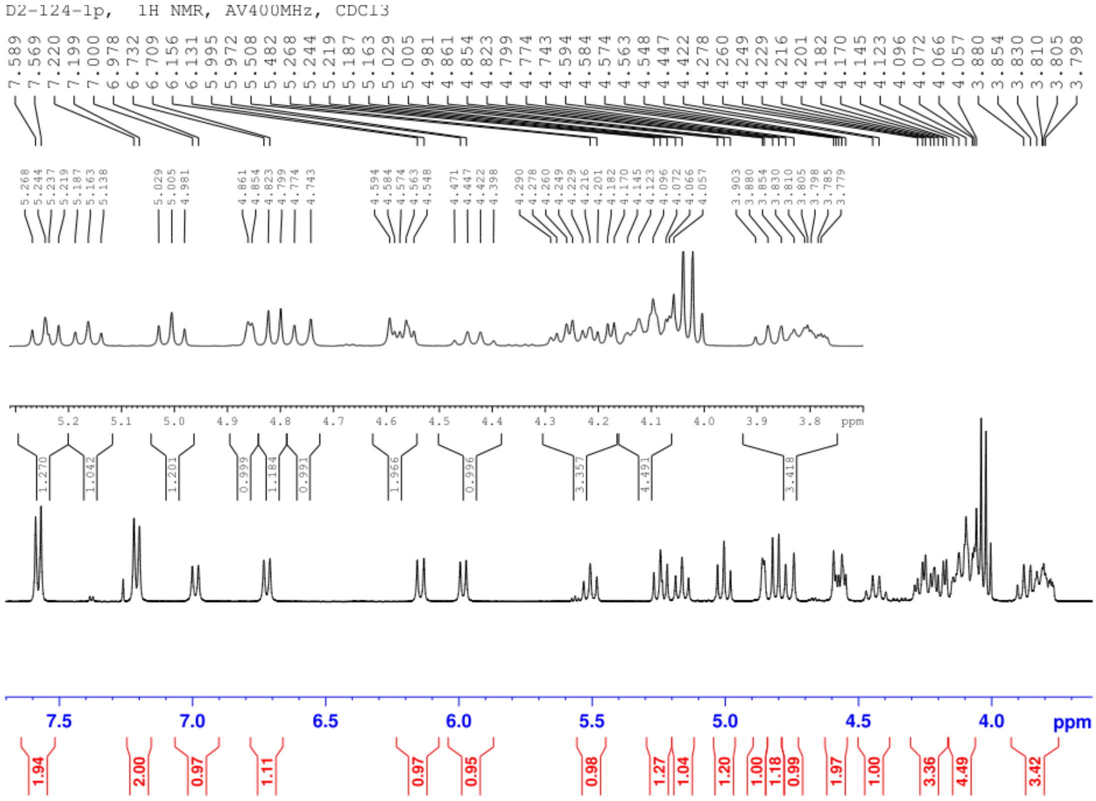
D2-124-1p, 1H NMR, AV400MHz, CDCl3

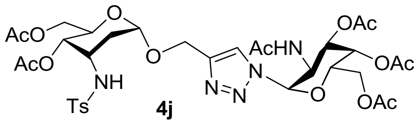

4j

D2-124-1p, 13C NMR, AV400MHz, CDCl3

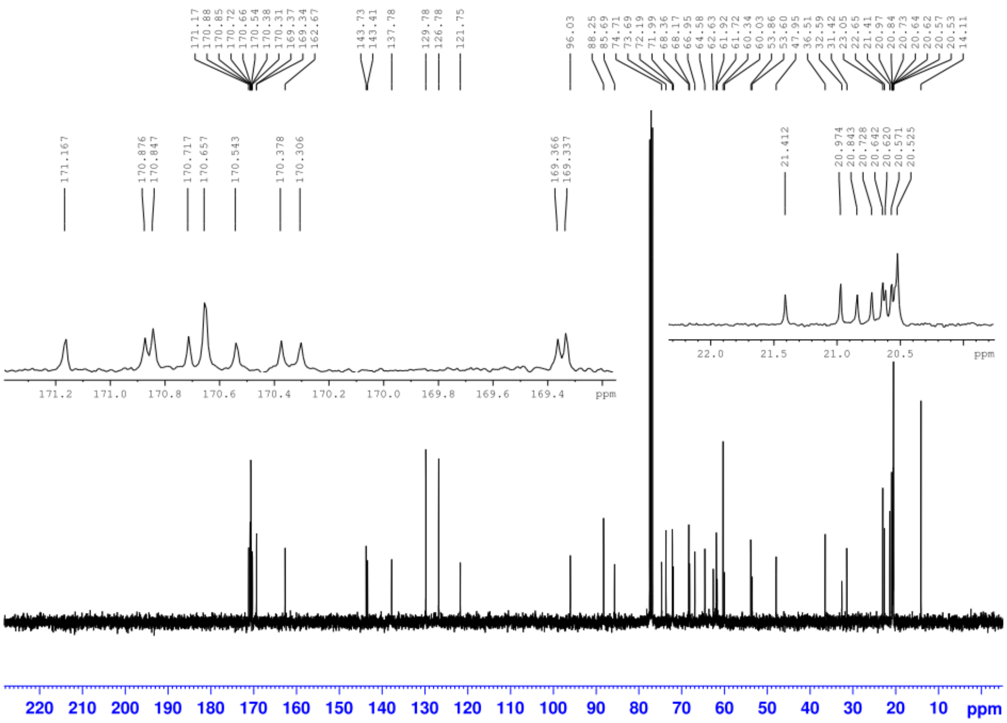

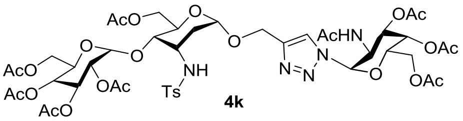

4k

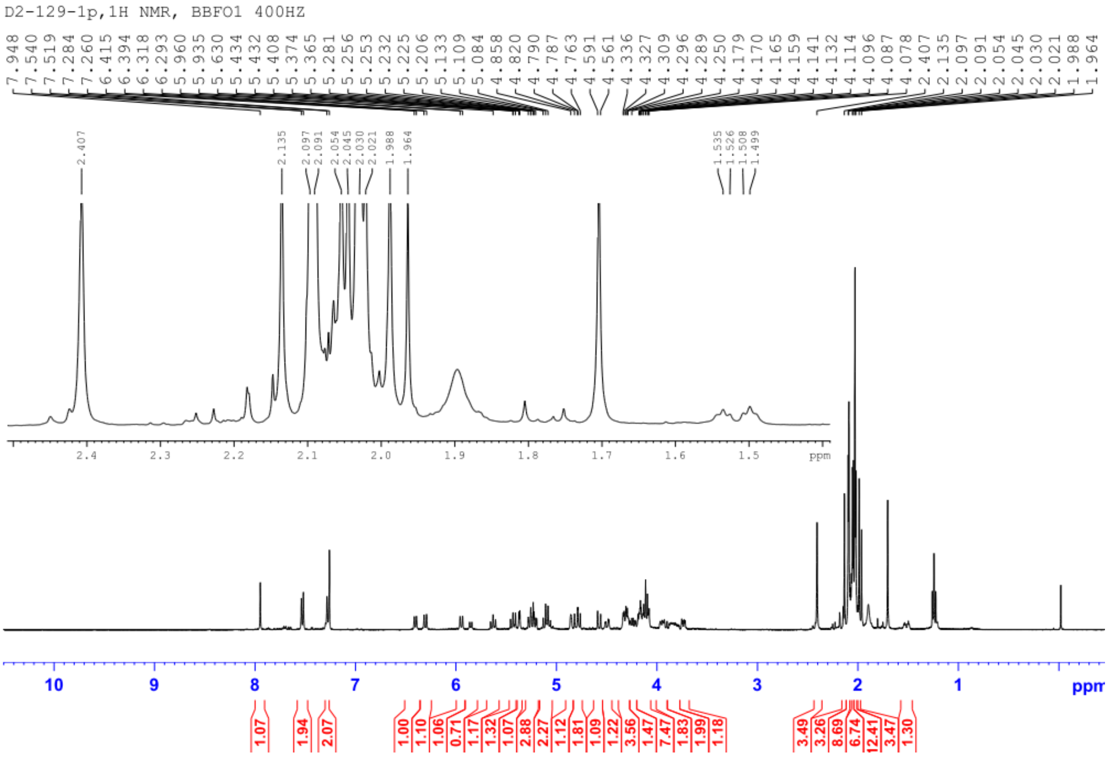

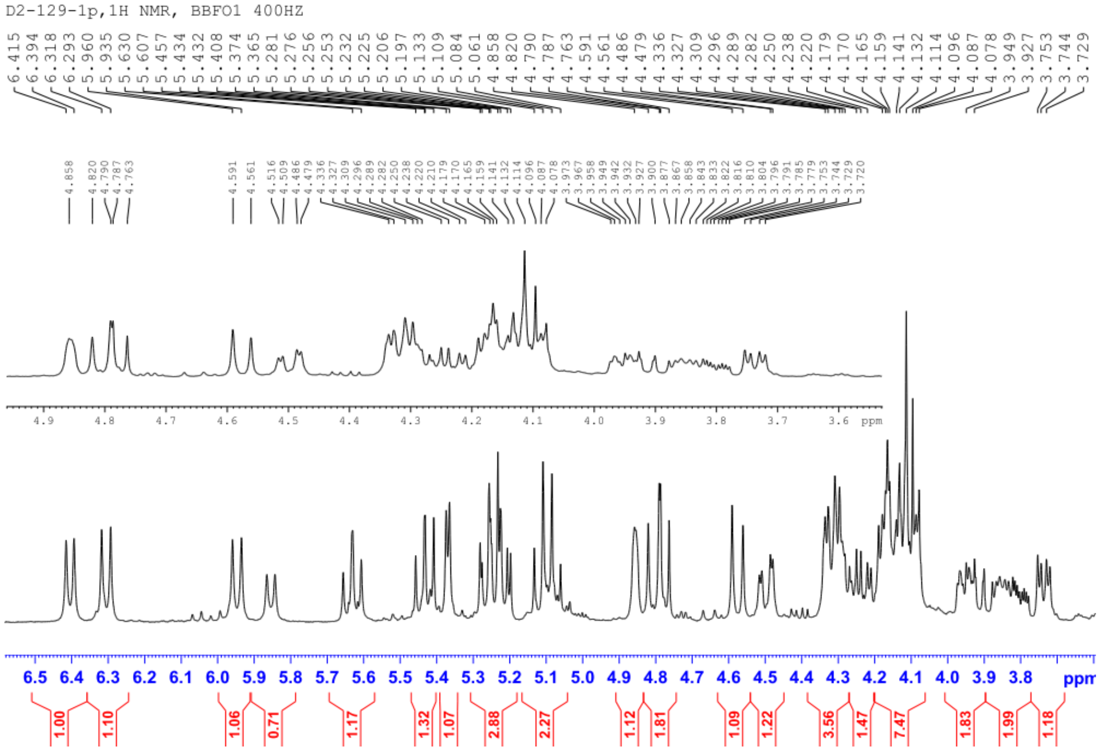

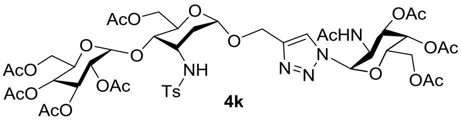

4k

D2-129-1p,13C NMR, BBFO1 400HZ

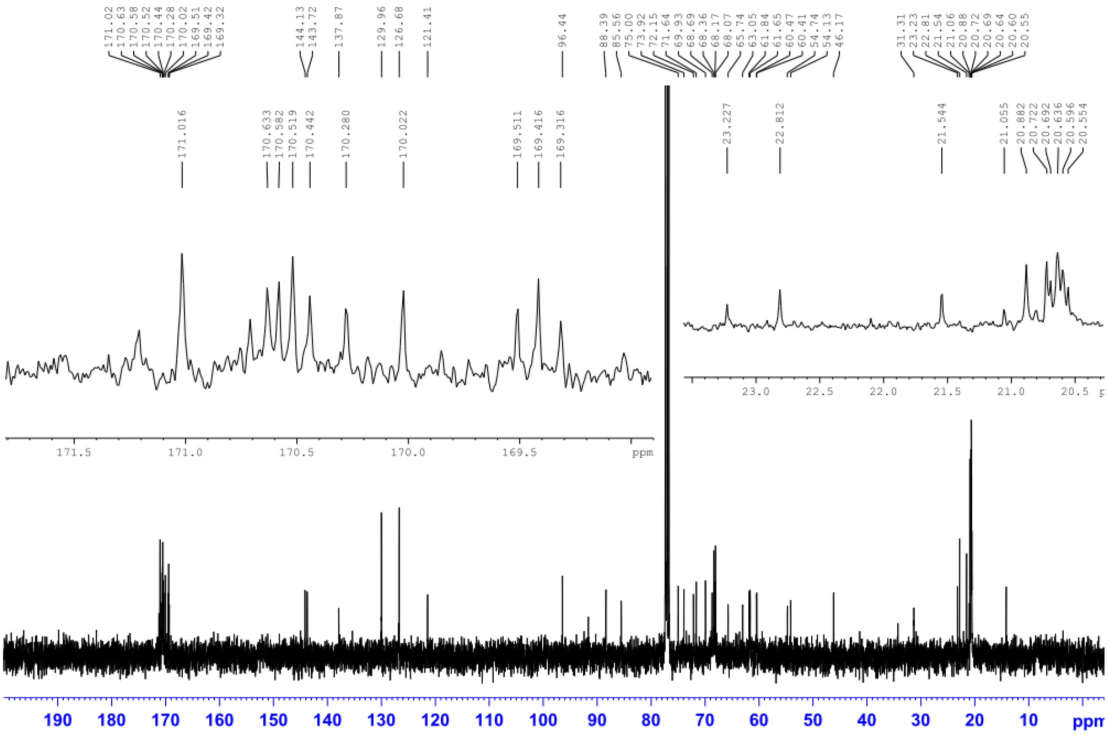

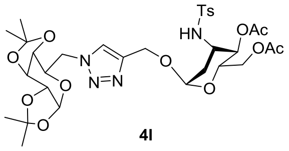

4l

D3-030-1p, 1H Av 400 MHz CDCl3, sep11

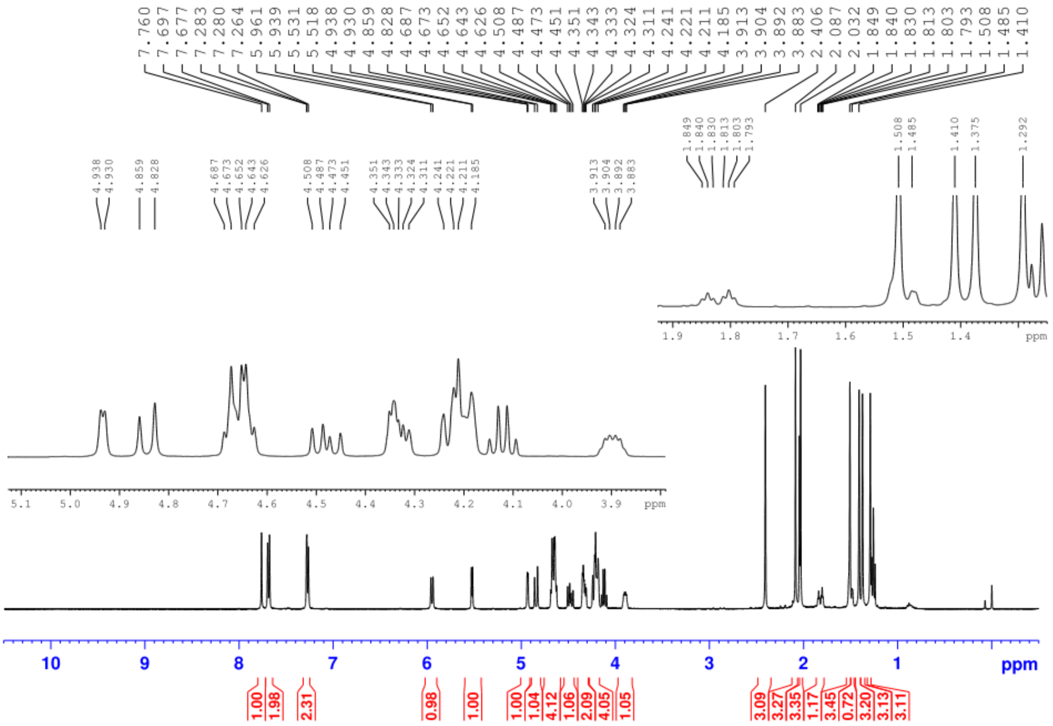

D3-030-1p, 13C Av 400 MHz CDCl3, sep11

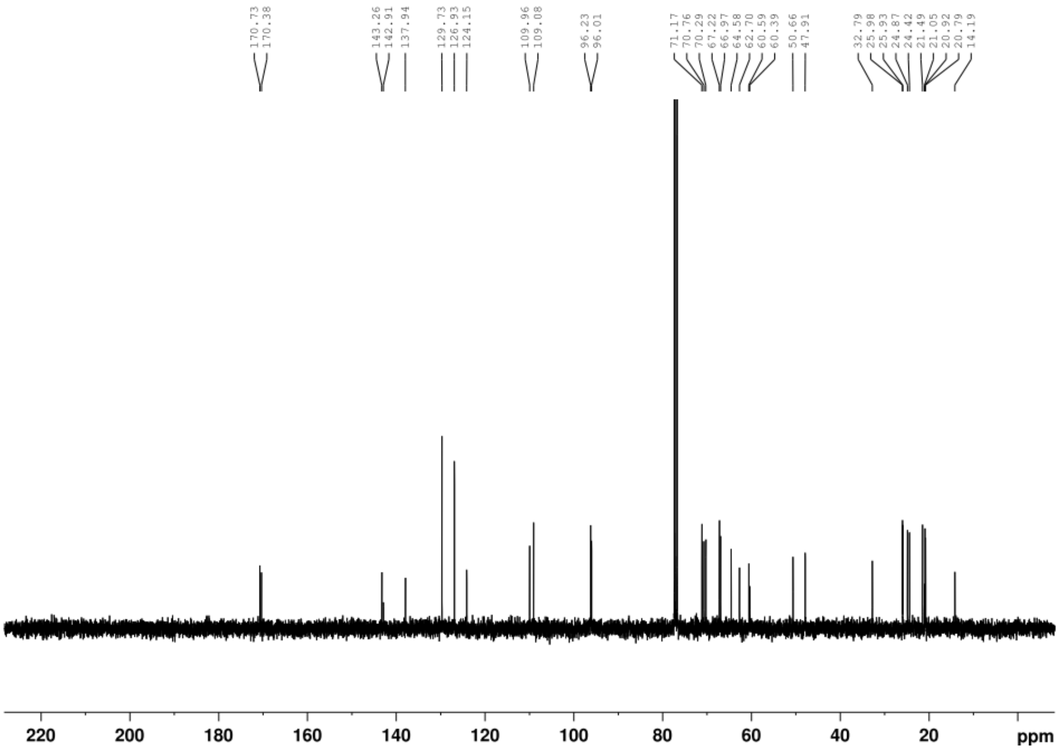

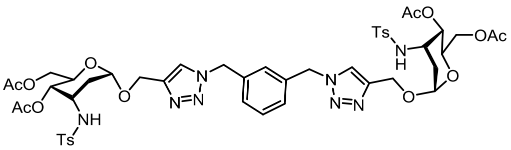

6a

D2-132-1p, 1HNMR, CDCl3, AV400MIHz

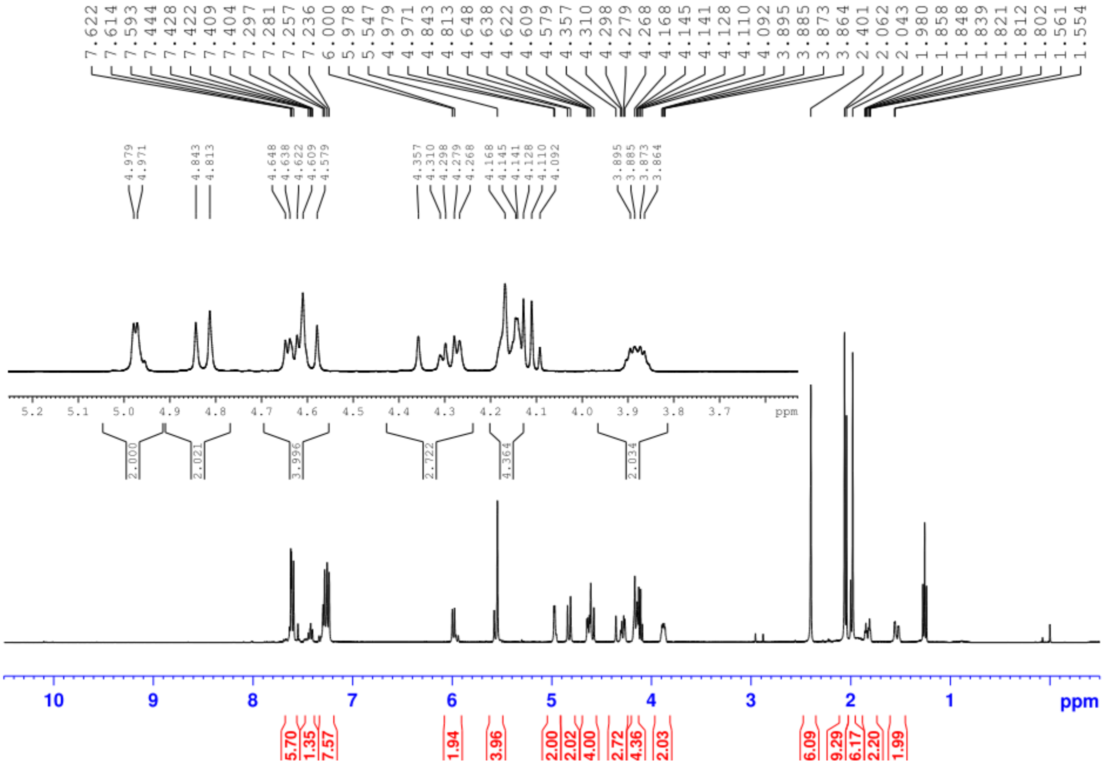

D2-132-1p, 13CNMR, CDCl3, AV400MIHz

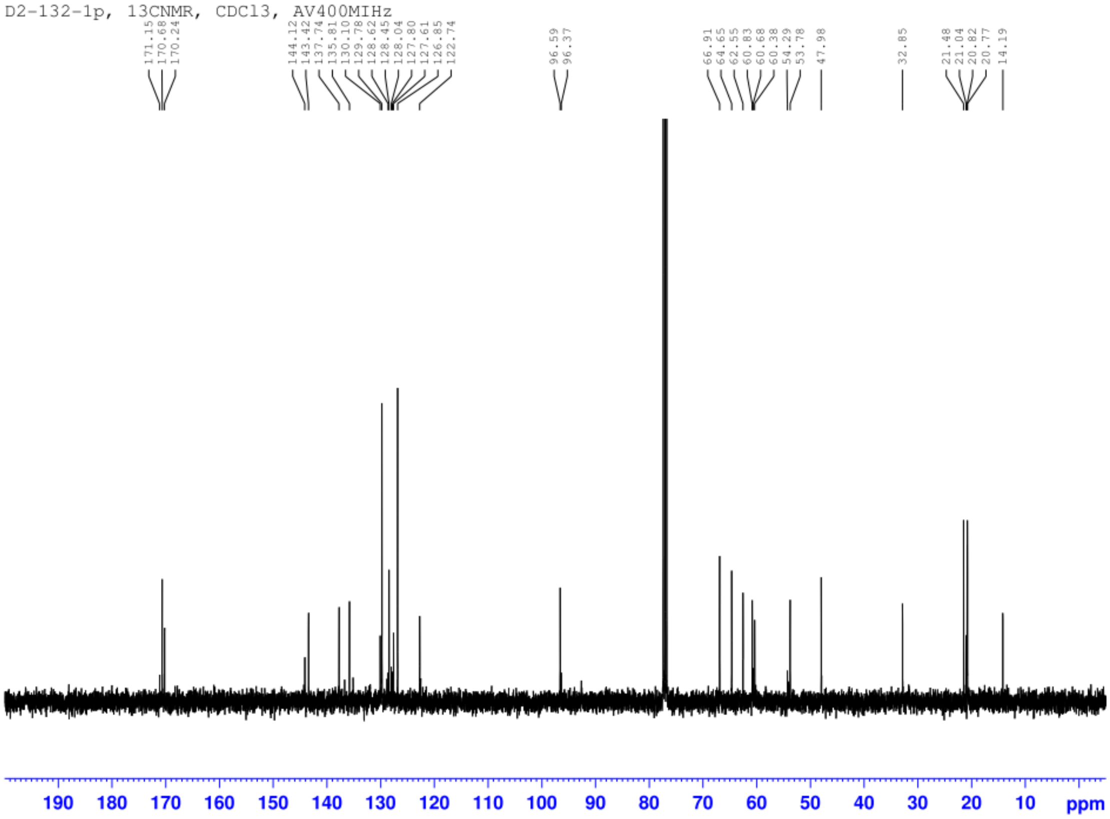

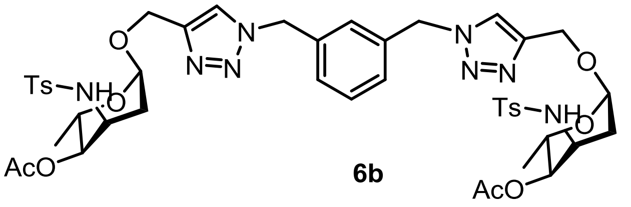
Ts
NH
O
N
N=N
AcO

6b

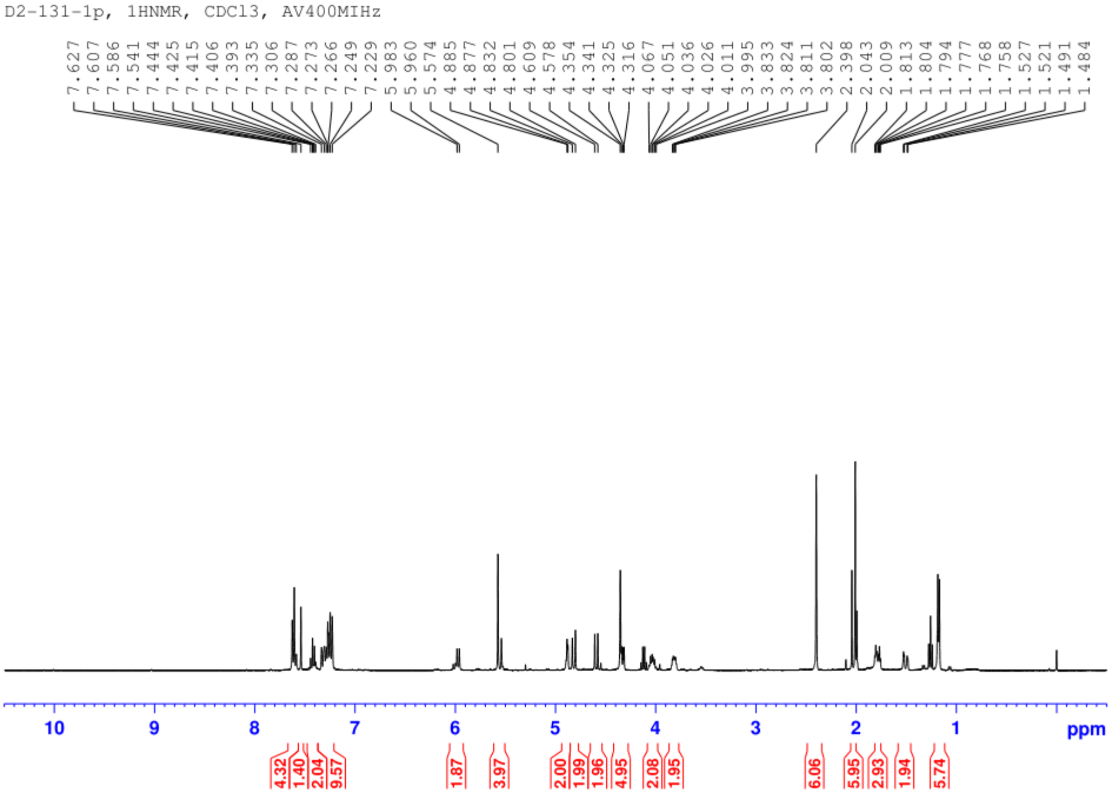
D2-131-1p, 1HNMR, CDCl3, AV400MIHz
7.627 7.607 7.586 7.541 7.444 7.425 7.415 7.406 7.393 7.335 7.306 7.287 7.273 7.266 7.249 7.229 5.983 5.960 5.574 4.885 4.877 4.832 4.801 4.609 4.578 4.354 4.341 4.325 4.316 4.067 4.051 4.036 4.026 4.011 3.995 3.833 3.824 3.811 3.802 2.398 2.043 2.009 1.813 1.804 1.794 1.777 1.768 1.758 1.527 1.521 1.491 1.484
10 9 8 7 6 5 4 3 2 1 ppm
4.32 1.40 2.04 9.57 1.87 3.97 2.00 1.99 1.96 4.95 2.08 1.95 6.06 5.95 2.93 1.94 5.74

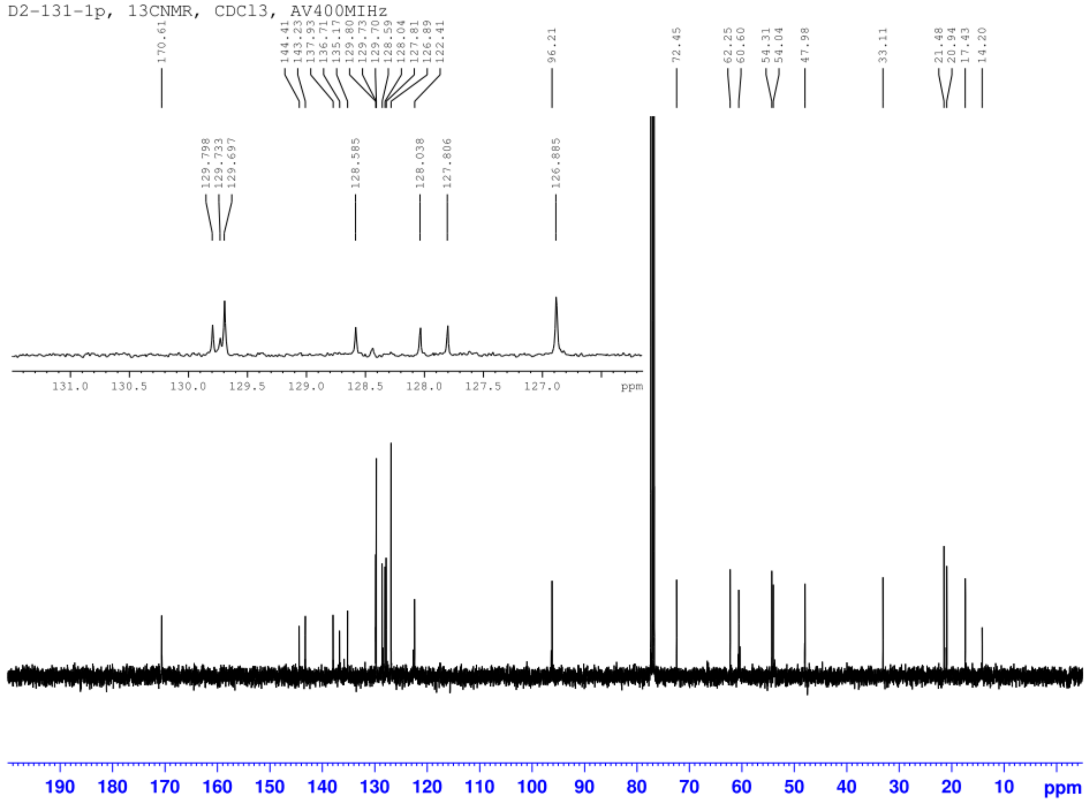
D2-131-1p, 13CNMR, CDCl3, AV400MIHz
170.61 144.41 143.23 137.93 136.71 135.17 129.80 129.73 129.70 128.59 128.04 127.81 126.89 122.41 96.21 72.45 62.25 60.60 54.31 54.04 47.98 33.11 21.48 20.94 17.43 14.20
129.798 129.733 129.697 128.585 128.038 127.806 126.885
131.0 130.5 130.0 129.5 129.0 128.5 128.0 127.5 127.0 ppm
190 180 170 160 150 140 130 120 110 100 90 80 70 60 50 40 30 20 10 ppm

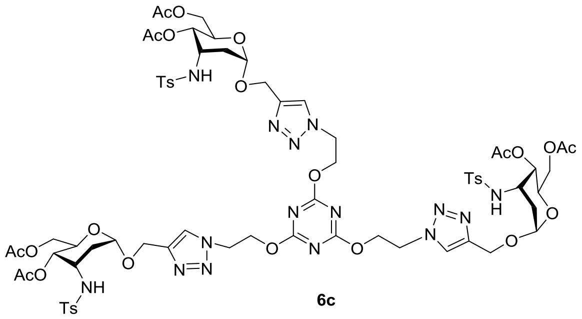
AcO
AcO
Ts
NH
O
N
OAc
H

6c

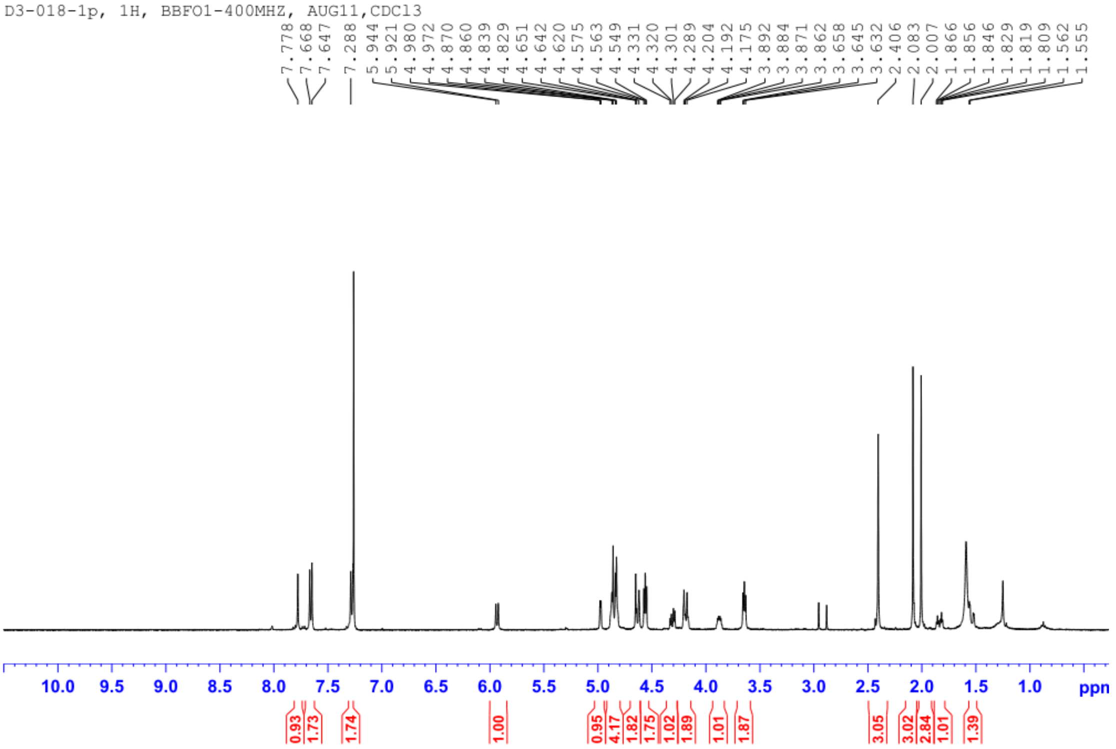
D3-018-1p, 1H, BBFO1-400MHZ, AUG11,CDCl3
7.778 7.668 7.647 7.288 5.944 5.921 4.980 4.972 4.870 4.860 4.839 4.829 4.651 4.642 4.620 4.575 4.563 4.549 4.331 4.320 4.301 4.289 4.204 4.192 4.175 3.892 3.884 3.871 3.862 3.658 3.645 3.632 2.406 2.083 2.007 1.866 1.856 1.846 1.829 1.819 1.809 1.562 1.555
0.93 1.73 1.74 1.00 0.95 4.17 1.82 1.75 1.02 1.89 1.01 1.87 3.05 3.02 2.84 1.01 1.39
10.0 9.5 9.0 8.5 8.0 7.5 7.0 6.5 6.0 5.5 5.0 4.5 4.0 3.5 3.0 2.5 2.0 1.5 1.0 ppm

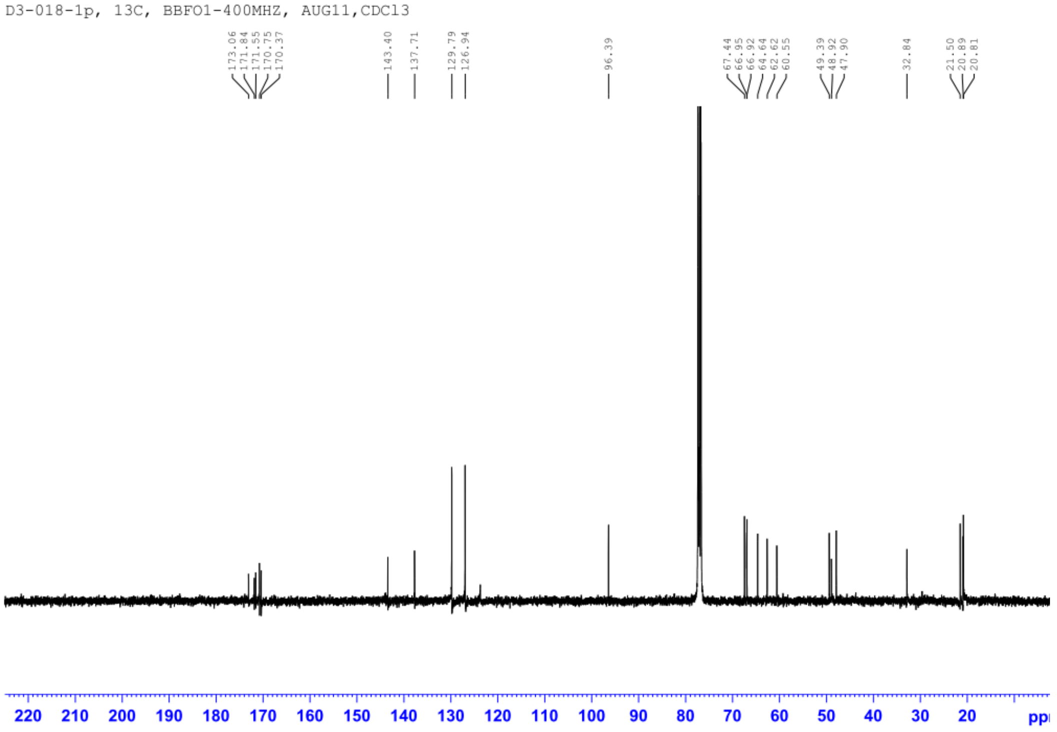
D3-018-1p, 13C, BBFO1-400MHZ, AUG11,CDCl3
173.06 171.84 171.55 170.75 170.37 143.40 137.71 129.79 126.94 96.39 67.44 66.95 66.92 64.64 62.62 60.55 49.39 48.92 47.90 32.84 21.50 20.89 20.81
220 210 200 190 180 170 160 150 140 130 120 110 100 90 80 70 60 50 40 30 20
